# Psychosocial Impact of COVID-19 on Intensive Care Unit Personnel: A Repeated Cross-Sectional Survey Assessment Before, During, and After the First Peak

**DOI:** 10.3390/healthcare14091154

**Published:** 2026-04-25

**Authors:** Nicholas C. Watson, Kathrine Kelly, Laura Krech, Alistair Chapman, Steffen Pounders, Matthew Armstrong, Charles J. Gibson, Gaby Iskander

**Affiliations:** 1 Department of Anesthesiology, Corewell Health, 100 Michigan St. NE, Grand Rapids, MI 49503, USA; matthew.armstrong@corewellhealth.org; 2 Trauma Research Institute, Corewell Health, MC 86-100 Michigan St. NE, Grand Rapids, MI 49503, USA; laura.krech@corewellhealth.org (L.K.); alistair.chapman@corewellhealth.org (A.C.); steffen.pounders@corewellhealth.org (S.P.); 3 Wellstar Summit Surgical, 590 Nancy Street, Marietta, GA 30060, USA; kelly.kathrine1@gmail.com; 4 Department of Surgery, Corewell Health, MC 250-100 Michigan St. NE, Grand Rapids, MI 49503, USA; charles.gibson@corewellhealth.org; 5 Department of Surgery, Valleywise Health Medical Center, 2601 E. Roosevelt St., Phoenix, AZ 85008, USA; giskande@hotmail.com

**Keywords:** intensive care unit, stress, COVID-19, burnout, compassion satisfaction, secondary traumatic stress, professional quality of life

## Abstract

**Highlights:**

**What are the main findings?**

- Intensive care unit workers reported high levels of compassion satisfaction, burnout, and secondary traumatic stress before, during and after the first peak of COVID-19.
- Motivation to work was associated with feeling protected by the government and hospital, while hesitation to work was associated with concern of becoming infected, feelings of isolation, and exhaustion.
- Gender, work role, work location, and unit subtype were associated with intensive care unit workers’ willingness to work.

**What are the implications of the main findings?**

- Intensive care unit worker motivation and hesitation to work are influenced by multiple factors that may represent targets to improve willingness to work during critically stressful conditions.

**Abstract:**

**Background/Objectives**: The coronavirus disease 2019 (COVID-19) pandemic presented significant psychosocial challenges to intensive care unit health care workers (ICU HCW). Prior studies typically used single cross-sectional samples to focus on elements of burnout and psychological stress. We sought to serially assess quality of life and willingness to work before, during, and after the first peak of COVID-19. **Methods**: Two survey instruments were prospectively administered at regular intervals to multidisciplinary ICU HCWs, initiating at the local onset of COVID-19 and ending 6 months after the first peak ICU census of COVID-19 patients. **Results**: ICU HCWs reported high levels of compassion satisfaction, burnout, and secondary traumatic stress before, during and after the first peak of COVID-19. Motivation to work declined, and hesitation to work increased from study initiation to the peak ICU census of COVID-19 patients. Hesitation to work was greater in female HCWs and cardiothoracic ICU HCWs. Motivation to work was higher in those working in operating rooms compared to those in the ICU. Concerns about becoming infected, feelings of isolation, and exhaustion were associated with high hesitation to work. Feeling protected by the government and hospital was associated with decreased hesitation and increased motivation to work. **Conclusions**: ICU HCWs experienced high levels of stress throughout the first year of COVID-19, while satisfaction with work remained high. Willingness to work was associated with gender, work location, ICU subtype, concerns about infection risk, feelings of exhaustion, and feelings of institutional protection. Because the study methodology precludes causal inference and low survey response rates indicate that findings should be interpreted with caution, these results are best viewed as hypothesis-generating for future work aimed at improving stress mitigation in ICU HCWs.

## 1. Introduction

### 1.1. Background

The coronavirus disease 2019 (COVID-19) pandemic presented significant challenges to the physical, mental, and social welfare of health care workers (HCWs) and their families. Concerns for HCW infection and death were apparent from the onset of the pandemic [1]. The World Health Organization estimated that between 1 January 2020 and 16 May 2021 there were 80,000 to 180,000 COVID-19-related HCW deaths [2]. While the cumulative number of confirmed COVID-19 deaths exceeded 7 million as of November 2024, the total HCW death toll remains unknown [3].

Additionally, the global pandemic intensified psychological strain in HCWs, a population with pre-existing elevated levels of work-related stress [4,5,6,7,8,9,10,11,12,13,14,15]. Many systematic reviews, scoping reviews, and meta-analyses demonstrated that HCWs had elevated levels of stress, anxiety, depression, sleep disturbances, cutaneous manifestations, burnout (BO), emotional exhaustion, depersonalization, post-traumatic stress, and fear at various time points in the pandemic [16,17,18,19,20,21,22,23,24,25,26,27,28]. Adverse psychological findings were typically more severe and more prevalent in women, nurses, and frontline workers compared to men, non-nurse HCWs, and non-frontline HCWs, respectively. The majority of cross-sectional studies included in these COVID-19 reviews captured data at a single time point. While significantly rarer, studies using longitudinal evaluations of HCW response demonstrated worsening BO from pre-pandemic to intra-pandemic while other markers such as stress, distress, and anxiety increased, decreased, or remained stable across intra-pandemic time points [7,8,11,29,30,31,32,33,34,35,36,37,38,39,40,41]. Furthermore, a subset of studies focused on intensive care unit (ICU) HCWs, showing high levels of BO, anxiety, and depression [7,8,31,40,42,43,44,45,46,47]. Exiguous studies examined ICU HCWs at serial peri-pandemic time points, demonstrating higher rates of BO during the pandemic compared to before the pandemic, increasing BO and depression across the second peak, and sustained psychological distress throughout the pandemic [7,8,31,40].

Despite the corpus of work highlighted above, several gaps remain in the literature surrounding the psychological response of ICU HCWs to COVID-19:

(1) Professional quality of life was incompletely assessed.
(2) Willingness to work and related factors were not directly assessed.
(3) Serial assessment of ICU HCWs was uncommon.

### 1.2. HCW Professional Quality of Life

The well-being of HCWs is linked to a multitude of factors. Studies aimed at disentangling the various causes, effects, and associations of HCW well-being have produced an array of theoretical constructs that commonly overlap, adopt, or subsume features [37,48,49,50,51,52,53,54,55,56,57,58]. Here we focus on professional quality of life as a psychosocial model that conceptualizes the overall well-being of a caregiver by considering both the positive and negative aspects of the work experience [53]. Operationally, the model incorporates three assessable dimensions: compassion satisfaction (CS), BO, and secondary traumatic stress (STS). CS, defined as the pleasure derived when one is able to do one’s work well, is associated with feelings of fulfillment, reward, achievement, happiness, enrichment, inspiration, energy, gratitude, and hope [53]. Negative aspects of work are framed as compassion fatigue, composed of BO and STS. BO is marked by exhaustion, frustration, anger and depression, while STS is driven by fear and work-related trauma [53]. CS has been shown to be protective against BO and STS [59,60,61].

We selected professional quality of life as a central element of our study precisely because it reflects both positive and negative aspects of HCWs’ experience. While negative factors such as BO and variables similar to STS were commonly assessed during the pandemic, CS was rarely evaluated. Prior isolated assessments of BO fail to provide a holistic picture of HCW well-being; however, contemporaneous measurement of CS, BO, and STS captures a more comprehensive perspective. Assessment of multiple time points opened the possibility for identifying temporal changes in each element of professional quality of life across the peri-peak period.

### 1.3. HCW Willingness to Work

Willingness to work is an expansion of specific elements of the professional quality of life framework. For example, CS includes the concept of “energy” for work, and compassion fatigue includes the concepts of exhaustion, frustration, and fear. Our evaluation of willingness to work was designed to assess work energy, exhaustion, frustration, fear and related factors specifically in the context of COVID-19. Willingness to work is a function of hesitation and motivation to work. Factors that increase motivation do not necessarily decrease hesitation and vice versa [62]. Thus, a separate analysis of motivation and hesitation is required. Research concerning the H1N1 influenza pandemic showed that some factors simultaneously increased both hesitation and motivation to work in HCWs [62]. The corpus of psychosocial studies focusing on negative effects and maladaptive responses during COVID-19 overlooks the value of dissecting the relationship between motivation and hesitation to work. The simple identification of factors that provoke hesitation to work does not ensure that removal or amelioration of these factors will yield the desired consequence of improved motivation to work. Understanding factors that motivate HCWs while separately assessing factors that discourage HCWs can assist in the comprehensive identification of targets for interventions to help HCWs in achieving personal and organizational work goals during future crises.

### 1.4. Theoretical Frameworks of Temporal Stress Response Relevant to HCW Professional Quality of Life

While the formal dimensions of professional quality of life have utility as research metrics, several complementary conceptual frameworks serve to explain temporal changes in HCW professional quality of life in response to sustained stress.

First, the general adaptation syndrome explains the body’s temporal stress response as an initial alarm reaction stage (immediate reaction to stressor marked by elevated cortisol and adrenaline), then resistance stage (stress hormones remain elevated while attempting to adapt to ongoing stress), and finally exhaustion stage (poor physical and mental health result from sustained stress, marked by fatigue, BO, depression, and anxiety) [63,64]. Further refinement of the general adaptation syndrome via the allostatic load model holds that biological stability is maintained by neural, neuroendocrine, and immune mechanistic adaptations to stress [65]. Repeated activation of allostatic systems (e.g., COVID-19) can lead to a dysregulated stress response over time, with a positive correlation between BO and allostatic overload demonstrated in HCWs [66]. Via modulation of BO, the allostatic load influences professional quality of life. While the links between physical and psychological health are well described, additional contemporary frameworks offer nuanced explanations that elaborate on psychological, social, and organizational factors.

Second, moral injury, defined as the distressing psychological, behavioral, social, and spiritual aftermath in response to acting or witnessing behaviors that violate an individual’s values and moral beliefs, was common among HCWs during COVID-19 [67,68]. Multiple specific sources of moral injury were identified during COVID-19: insufficient resources (especially personal protective equipment), imbalance of patient care duties with personal and family safety, inequality of health care delivery, role conflict between patient autonomy and regulatory/institutional parameters, low rewards, and high emotional demands [69,70,71,72]. Moral distress was positively correlated with emotional exhaustion, depersonalization, BO, and STS, thus directly affecting components of professional quality of life [73,74]. Willingness to work includes variables identified with moral injury, including work energy, exhaustion, frustration, and fear. A vicious cycle of moral injury causing staff turnover and altered patient care, which then contributed to further moral injury, was proposed as an explanation for the worsening trajectory of HCW quality of life [71].

Third, conservation of resources theory posits that the perceived or actual loss of objects, conditions, personal characteristics, and energy contributes to HCW BO and STS [49,75]. Similarly, the resource depletion model proposes that ongoing or accumulating stress drains coping capacity over time [76]. The variables for evaluating willingness to work include HCW resources for stress management [62]. Conservation of resources theory frames temporal decline in quality of life as a resource “loss spiral”, such that initial stressors deplete resources, which leaves HCWs with insufficient resources to respond to future stress, thus compounding decline of well-being [77].

Fourth, a growing body of evidence demonstrates the important role of emotion regulation for HCW well-being. Emotion regulation, a central construct in differentiating individual stress responses, is a coping strategy marked by the management of emotional experiences and behavioral responses to specific stressful conditions without being overwhelmed by feelings [78,79]. Similar, yet distinct, is the construct of psychological flexibility, which describes an expansive range of adaptive strategies for individual responses that cover differing situational demands, shifting mindsets and behaviors, maintenance of life balance, and value-based behaviors [80]. Emotion regulation and psychological flexibility have been associated with professional quality of life. Adaptive emotion regulation was associated with lower BO rates, thus linking emotion regulation to professional quality of life [81,82]. Appraisal of work as a cognitive challenge (vis-a-vis threat) directly and positively impacted motivation in the workplace [83,84]. Similarly, components of psychological flexibility such as higher resilience, positive affect, emotional/social support, and lower perceived stress contributed to elevated professional quality of life [60,84,85,86,87,88,89,90]. Increased psychological flexibility corresponded with increased work engagement and partially mediated the effects of job satisfaction on mental well-being [58,90].

Contrastingly, maladaptive responses of poor or absent emotion regulation lead to undesirable effects on professional quality of life. Perception of higher job demand increased self-perceived stress and decreased self-perceived quality of life [87]. Higher difficulties in emotion regulation predicted greater psychological inflexibility, which, in turn, predicted higher perceived stress [79]. Contact with patients with COVID-19 predicted increased use of emotion suppression strategies, which then further increased stress in HCWs [91,92,93]. Reliance on other emotion regulation maladaptive strategies such as experiential avoidance and expressive suppression was associated with high psychological impact during COVID-19 [67]. Higher perceived stress and threat cognitive appraisal were associated with lower motivation to work [58,68]. Fatigue (an element of BO) and STS were associated with higher levels of emotional dysregulation and reduced metacognitive abilities [62]. Fatigue appeared to also depend on a tendency toward disengagement from the use of avoidance and nonacceptance of responses to emotional distress [62].

Thus, with the above conceptual framing to support the importance of HCW professional quality of life and willingness to work during the COVID-19 pandemic, we undertook the current study.

### 1.5. Study Objectives

The objective of our study was to assess the temporal variation in the psychosocial impact of the COVID-19 pandemic on multidisciplinary ICU HCWs in all subtypes of ICUs at a tertiary referral center. The specific aims of this study were to: (1) measure professional quality of life (compassion satisfaction, burnout, and secondary traumatic stress) before, during, and after the first peak ICU census of COVID-19 cases; (2) assess willingness to work (motivation to work and hesitation to work) before, during, and after the first peak ICU census of COVID-19 cases; and (3) evaluate factors associated with willingness to work. To our knowledge, this is the first study to prospectively measure ICU HCW psychosocial response to the COVID-19 pandemic at the distinct time points of pre-peak, first peak, and post-peak. Similarly, this study is unique in serially evaluating compassion satisfaction, motivation to work, and hesitation to work during COVID-19 in a multidisciplinary ICU sample.

## 2. Materials and Methods

A consensus-based checklist for reporting of survey studies was followed (see Supplement S1) [94]. The study protocol (2020-165 SH/HDVCH) was submitted to our Institutional Review Board and approved on 17 April 2020. The inclusion criteria were all HCWs who provided care for adult ICU patients at Spectrum Health during the study period. Administering the survey to ICU HCWs was designed to have the sample population reflect the target audience for application of the study findings, thus limiting sample bias. Spectrum Health is a tertiary medical center consisting of 1107 total beds, 116 ICU beds, and a large geographic catchment area. The hospital system has multiple sub-specialty ICUs, including medical, surgical, cardiothoracic, neurologic, and mixed medical/surgical. ICU HCWs included defined job roles of physician, advanced practice provider, nurse anesthetist, nurse, nursing technician, nursing assistant, and pharmacist. Advanced practice providers included nurse practitioners and physician assistants. Physicians included medical intensivists, pulmonologists, surgical intensivists, surgeons, anesthesiologists, and neuroscience intensivists. Potential study participants were identified by hospital electronic mail groups corresponding to work location. Duplicate addresses were eliminated.

### 2.1. Survey Development and Content

Two survey instruments were used: (1) the Professional Quality of Life Measure Version 5 (ProQOL) and (2) the Work-Related Stress Survey (WRSS) (see Supplements S2 and S3, respectively).

The ProQOL is a list of 30 statements concerning helping other people with a 5-point Likert-style response scale (1 never, 2 rarely, 3 sometimes, 4 often, 5 very often) [53]. ProQOL is intended for any “helper”, expressly including health care professionals, and is a commonly used research measure of the positive and negative effects of working with people who have experienced extremely stressful events. The ProQOL has been used in more than 600 studies, including several evaluating HCWs during the COVID-19 pandemic [32,95,96,97,98,99]. ProQOL assesses 3 subscales: CS, BO, and STS. We obtained formal permission to use the ProQOL Version 5 from the Center for Victims of Torture [100]. No modifications were made to the ProQOL. Each ProQOL administration also asked participants to identify sociodemographic data, including primary work location in the 2 weeks preceding the survey, age, gender identity, job type, household pre-tax income, whether they had children at home, whether they provided elder or dependent care (independent of their employment), full- or part-time employment, marital status, self-described health status, and years of experience in their medical career.

The WRSS is a list of 19 items concerning stress, emotions, hesitancy to work, and motivation to work in the context of the COVID-19 pandemic. It uses a 4-point Likert-style response scale (0 never, 1 rarely, 2 sometimes, 3 always). Free-text responses are permitted for items “please identify any stress reduction techniques you have used in the past week” and “additional comments”. Each WRSS administration also asked the participants to identify sociodemographic data, including primary work location in the 2 weeks preceding the survey, age, gender identity, and job type. The items in the WRSS were modeled on a questionnaire used during the 2009 H1N1 pandemic in Japan with modifications to enhance its relevance to COVID-19 [62]. The original questionnaire, available for unrestricted use under the terms of the Creative Commons Attribution license, contained elements from studies on severe acute respiratory syndrome (SARS) and hypothetical infectious pandemics [101,102,103,104,105].

Unlike the ProQOL, the WRSS has not been used in hundreds of studies. For this reason we sought to limit threats to validity and reliability of the WRSS. The authors performed a literature review to broadly identify recurrent themes and terminology used in psychosocial studies of HCWs during stressful events. These themes and terminologies were generally consistent with the original 2009 questionnaire, suggesting high content validity and reliability of the items, so no edits were made on this basis. The authors who were ICU HCWs performed expert validation to ensure that the content and scope of the items adequately addressed the goals of the study. The threat of poor question format and the risk of response bias from the question ordering effect were mitigated by checking items against published lists of common pitfalls [106,107]. Initial modifications to the original questionnaire focused on COVID-19-specific language and minor edits in syntax. Variation in wording was assured to limit the risk of habituation bias. Cognitive interviews were performed wherein HCWs (n = 10) representing the intended survey recipients were individually asked to state out loud in their own words what each survey item stated/asked while an author listened. The purpose of cognitive interviews was to ensure that the items were being interpreted as intended, a maneuver to increase reliability. A few minor edits were made based on this process.

Pretesting was performed by releasing the final version of each survey to the authors. No issues were encountered with the logistics of sending and completing the surveys. Initial responses demonstrated that the survey links and automated data collection system functioned appropriately. Anecdotally reported time to complete each survey was less than 5 min. The threat of intervening events affecting survey responses within each testing point was ameliorated by having the surveys available for a limited time after solicitation of participants and asking respondents to frame their answers in the context of providing care over the past week (WRSS) or past 2 weeks (ProQOL). The risk of instrumentation bias was eliminated by releasing only the final version of each survey and making no subsequent changes during the time the surveys were available to participants. Non-response bias was not measured or controlled because of the anonymous nature of the surveys.

### 2.2. Survey Administration

A repeated cross-sectional survey design was used. All participation was voluntary and anonymous. Potential study participants received interval survey invitations via work electronic mail. Each electronic correspondence included a message explaining the study’s purpose and risks and offered the recipient the opportunity to anonymously opt out of receiving further invitations. An internet link unique to each survey and unique to each respondent was provided with every survey invitation. Study participants signaled agreement with informed consent by accessing their individual survey link. Study data were confidentially collected and managed using REDCap electronic data capture tools hosted at Spectrum Health [108,109]. Study data confidentiality and correspondence with survey recipients were managed by an institutional “honest broker” who functioned as a neutral intermediary not otherwise involved with the study. The honest broker assured that subjects’ personal identifiable data and survey responses were maintained separately [110].

**Figure 1:**
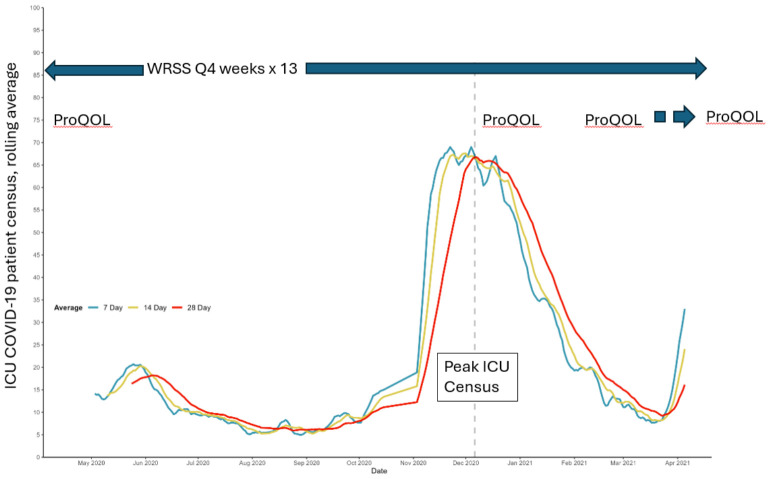
The study period mapped in the context of the ICU COVID-19-positive patient census. The ProQOL was administered at 4 time points: study initiation (20 April 2020), peak ICU census of COVID-19 patients (23 December 2020), 3 months post-peak (23 March 2021), and 6 months post-peak (21 July 2021). The WRSS was administered 13 times, starting on 27 April 2020 and every 4 weeks after. Abbreviations: ProQOL—Professional Quality of Life Measure Version 5. WRSS—Work-Related Stress Survey.

The World Health Organization declared the novel coronavirus outbreak a global pandemic on 11 March 2022 [111]. The first COVID-19 death at our hospital was on 21 March 2020 [112]. Accordingly, the first surveys were sent in April 2020. Figure 1 displays the study period in the context of the COVID-19-positive patient census within ICUs at our hospital system. The ProQOL was administered at 4 time points: study initiation (20 April 2020), peak ICU census of COVID-19 patients (23 December 2020), 3 months post-peak (23 March 2021), and 6 months post-peak (21 July 2021). The ProQOL was available to the recipients for 30 days after the solicitation email was sent.

The WRSS was administered 13 times over approximately one year, starting at study initiation (27 April 2020) and every 4 weeks thereafter. The WRSS was available to recipients for 10 days after the solicitation email was sent.

The census criteria were the daily count of patients admitted to an ICU who had a positive test result for COVID-19 polymerase chain reaction during admission. The peak census was estimated contemporaneously in order to time the ProQOL administration at peak. Following a 4-week steady rise in the ICU census of COVID-19 patients, the 7-day and 14-day rolling average census declined for 10 consecutive days, and the 28-day rolling average reached a plateau in December 2020. This time point was determined to be the peak.

### 2.3. Statistical Methods

Statistical analyses for the ProQOL were generated using SAS (SAS Enterprise Guide software, Version 7.1, SAS Institute Inc, Cary, NC, USA). Sociodemographic responses were calculated as a count for every respondent to one or more ProQOL survey questions over the study period. Sociodemographic data from only the first ProQOL survey each individual responded to were used in the tally, regardless of the total number of surveys completed. The ProQOL item responses were scored and scaled according to the Concise ProQOL Manual [53]. Only the ProQOL surveys with all items completed were accepted for analysis. Items 1, 4, 15, 17, and 29 were reversed, items were summed by subscale (CS, BO, STS), raw scores were converted to Z-scores, and Z-scores were converted to t-scores with the raw score mean = 50 and the raw score standard deviation = 10. Cut scores were set around the 25th and 75th percentiles based on the ProQOL databank and categorized as “low” (lower quartile), “moderate” (interquartile), and “high” (upper quartile). ProQOL responses were calculated as count and percentage of respondents per low/moderate/high category for each subscale for each time point at which the ProQOL was administered. Correlational analysis between the ProQOL subscales at each time point was performed using Kendall’s Tau. The ProQOL repeated measures were analyzed using a generalized estimating equation, assuming proportional odds.

Statistical analyses for the WRSS were also generated using SAS (SAS Enterprise Guide software, Version 7.1, SAS Institute Inc, Cary, NC, USA). WRSS responses were calculated as a count and percentage. WRSS with one or more items incomplete were accepted for analysis, with only the completed items counting toward the tally. Motivation to work and hesitation to work item responses were dichotomized into “low” (score of 0 “never” or 1 “rarely”) and “high” (score of 2 “sometimes” or 3 “always”). The percentages of “high” scores of motivation and hesitation were compared between study initiation and peak ICU census using the Chi-Square test. The percentages of “always” responses for motivation to work and hesitation to work were compared between study initiation and peak ICU census using the Chi-Square test.

Similarly, responses to the stress-related items were dichotomized into weak responses (score of 0 “never” or 1 “rarely”) and strong responses (score of 2 “sometimes” or 3 “always”). Univariate (U) and multivariate (M) logistic regression models (adjusted for age, gender, job and work location) were used to compute odds ratios (OR) and confidence intervals (CI) to analyze the association of demographic variables and strong responses to stress-related items with “high” motivation to work and “high” hesitation to work at two time points: the initial WRSS administered at study start and the WRSS administered at peak ICU census of COVID-19 patients. Statistical significance was determined at *p* < 0.05. Strong response counts to selected stress-related items were plotted across the study period (13 WRSS administrations in total) for the visualization of trends in comparison to a plot of ICU census of COVID-19 patients over the study period. Finally, WRSS free-text responses were tallied for frequency and represented with a word cloud [113].

## 3. Results

### 3.1. ProQOL Results

The ProQOL was sent to 621 (study initiation), 575 (peak census), 581 (3 months post-peak) and 610 (6 months post-peak) recipients. Response rates were n (%): 205 (33%) at study initiation, 92 (16%) at peak census, 64 (11%) at 3 months post-peak, and 61 (10%) at 6 months post-peak. The net response for all four ProQOL surveys combined was 422 (18%). While most survey recipients received all four surveys, the number of survey recipients at each time point varied due to changes in the number of ICU HCWs over the course of the study. There were 278 unique individual respondents to one or more ProQOL surveys. All surveys received were complete. Of the 278 respondents, most had a female gender identity (67%), were 30–39 years old (40%), were married (76%), and had non-adult dependent children (62%). Most respondents had a self-described health status of “good” (32%) or “very good” (46%). Income levels were distributed across a broad range. The most common job role was nurse (59%), followed by physician (21%) and advanced practice provider (9%). The primary work location was distributed across all the possible work locations. The detailed characteristics of ProQOL respondents are shown in Supplement S4.

CS scores were “high” for 81%, 78%, 83%, and 74% of respondents and “moderate” for 18%, 22%, 14%, and 21% of respondents on initial, peak census, 3-month post-peak, and 6-month post-peak surveys, respectively. Between 0% and 5% of respondents scored “low” for compassion satisfaction on each survey.

BO scores were “high” for 83%, 74%, 80%, and 79% of respondents on initial, peak census, 3-month post-peak, and 6-month post-peak surveys, respectively. Every remaining respondent scored “moderate” for burnout on each survey. No respondents scored “low” for burnout on any survey.

STS scores were “high” for 81%, 75%, 72%, and 70% of respondents on initial, peak census, 3-month post-peak, and 6-month post-peak surveys, respectively. Every remaining respondent scored “moderate” for STS on each survey. No respondents scored “low” for secondary traumatic stress on any survey. The ProQOL scores for CS, BO, and STS are shown in Table 1.

**Figure 2:**
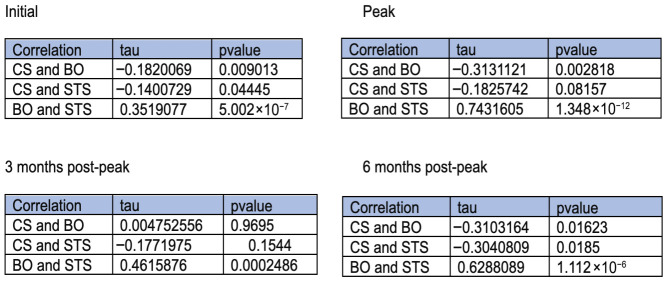
Kendall Tau correlation between ProQOL subscales at each study time point. Abbreviations: CS—compassion satisfaction, BO—burnout, STS—secondary traumatic stress.

Correlational analysis between ProQOL subscales at study initiation demonstrated a weak negative association between CS and BO, a weak negative association between CS and STS, and a moderate association between BO and STS. At peak ICU census of COVID-19 patients, a weak negative association between CS and STS, a moderately weak association between CS and BO, and a strong association between BO and STS were noted. At 3 months post-peak, there was almost no association between CS and BO, a weak negative association between CS and STS, and a moderate association between BO and STS. At 6 months post-peak, there was a moderately negative association between CS and BO, a moderately negative association between CS and STS, and a strong association between BO and STS. The details of the ProQOL correlational analysis are shown in Figure 2.

**Figure 3:**
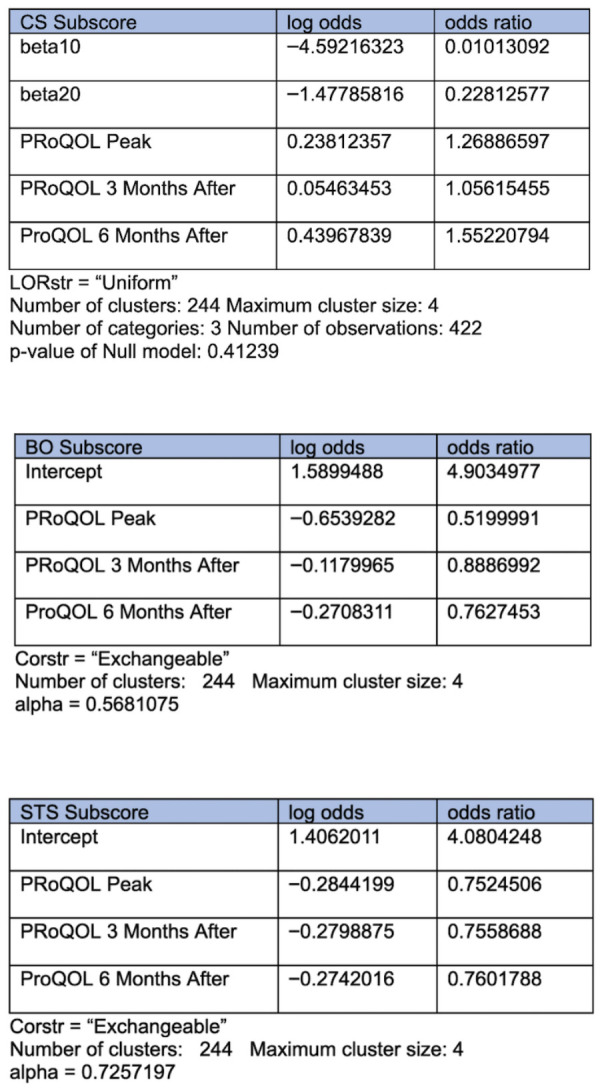
ProQOL subscales repeated measures analyzed using generalized estimating equation. Abbreviations: ProQOL—Professional Quality of Life Measure 5, CS—compassion satisfaction, BO—burnout, STS—secondary traumatic stress.

The ProQOL repeated measures were analyzed using a generalized estimating equation, assuming proportional odds. Details of this analysis are shown in Figure 3. Compared to the study initiation time point, the odds of the CS score falling in a higher category increase across the subsequent time points averaged across all respondents. Compared to the study initiation time point, each subsequent time point decreases the odds of the BO response falling into the “High” category vs. the “Moderate” category by ~48%, ~12%, and ~24%, respectively. Finally, compared to the study initiation time point, each subsequent time point decreases the odds of the STS response falling into the “High” category vs. the “Moderate” category by ~25%, ~24%, and ~24%, respectively.

### 3.2. WRSS Results

The WRSS was sent to 639 recipients at study initiation and had a gradually declining number of recipients over the study period, concluding with 558 recipients. While most survey recipients received all thirteen WRSS, the number of survey recipients at each time point varied due to changes in the number of ICU HCWs over the course of the study. Response rates were n (%): 173 (27%) at study initiation, 70 (12%) at peak ICU census, and 32 (5.7%) for the final WRSS. The net response for all thirteen WRSS combined was 1083 (14%). The WRSS response rates are detailed in Supplement S5. There were 278 unique individual respondents to one or more WRSS over the course of the study. Not all respondents submitted responses to all items; thus, the denominator for response calculation differs between some items. Of the 278 individual respondents, most were female (66%) and 30–39 years old (39%). The most common job role was nurse (49%), followed by physician (21%) and advanced practice provider (20%). The primary work location was distributed across possible work locations. WRSS aggregate respondent characteristics are shown in Supplement S6.

#### 3.2.1. Motivation to Work: Demographics

At study initiation, motivation to work was “high” in 151 (88%) respondents. This was subdivided as “sometimes motivated” 91 (53%) and “always motivated” 60 (35%). At study initiation, there were no statistically significant associations between motivation to work and age, gender identity, job role, or primary work location. Notably, participants in the age 60–69 years group reported 100% high motivation at study initiation.

**Figure 4:**
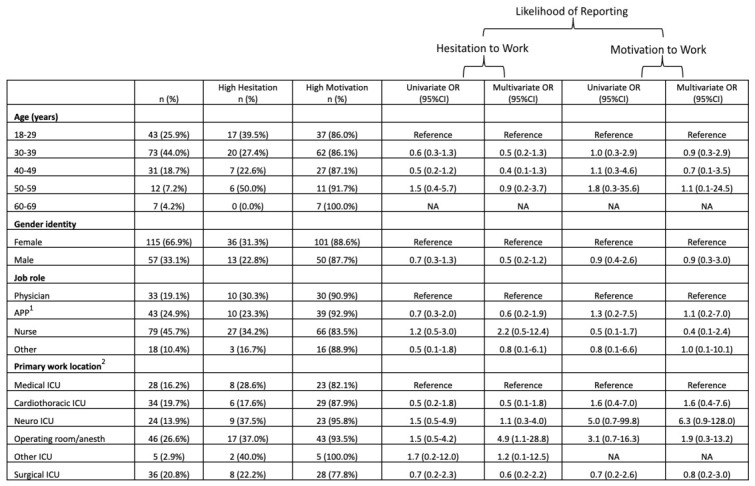
Association of demographic characteristics and likelihood of reporting hesitation and motivation to work at study initiation. Abbreviations: Uni—univariate. Multi—multivariate logistic regression models adjusted for age, gender, job and work location. OR—odds ratio. Notes: NA—Odds ratio values not reported due to small sample size or extreme values. 1—Advanced practice provider, defined as a nurse practitioner, certified registered nurse anesthetist, anesthesia assistant, or physician assistant. 2—Primary work location in the past two weeks. Operating room/anesth—primary work location of operating room or anesthetizing sites.

**Figure 5:**
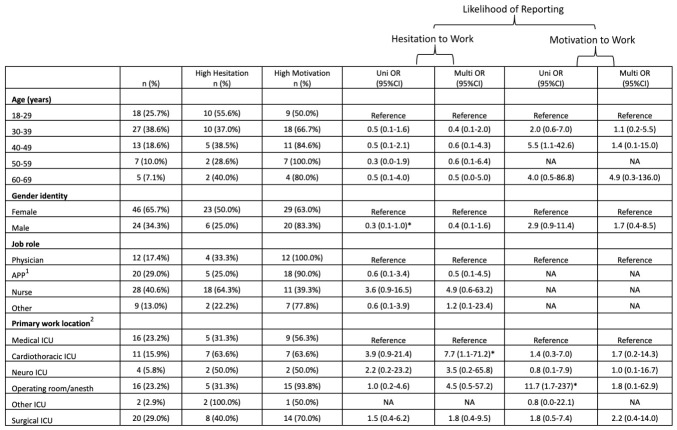
Association of demographic characteristics and likelihood of reporting hesitation and motivation to work at peak ICU census of COVID-19 patients. Abbreviations: Uni—univariate. Multi—multivariate logistic regression models adjusted for age, gender, job and work location. OR—odds ratio. Notes: * Denotes statistically significant value. NA—Odds ratio values not reported due to small sample size or extreme values. 1—Advanced practice provider, defined as a nurse practitioner, certified registered nurse anesthetist, anesthesia assistant, or physician assistant. 2—Primary work location in the past two weeks. Operating room/anesth—primary work location of operating room or anesthetizing sites.

At peak ICU census, motivation to work was “high” in 49 (70%) of respondents. This was subdivided as “sometimes motivated” 31 (44%) and “always motivated” 18 (26%). At peak ICU census, HCWs with primary work location of operating room/anesthetizing sites were more likely to report high motivation to work compared to the reference group of HCWs with primary work location of medical ICU (univariate (U) *p* = 0.033). At peak ICU census there were no statistically significant associations between motivation to work and age, gender identity, or job role. The percentage of respondents with “high” motivation to work was greater at study initiation compared to peak (*p* = 0.0009), whereas there was no difference in the percentage of people reporting “always motivated” (*p* = 0.1664). The associations of demographic characteristics and the likelihood of reporting motivation to work are shown in Figure 4 and Figure 5.

#### 3.2.2. Motivation to Work: Stress-Related Items

At study initiation, strong responses to multiple stress-related items were associated with a greater likelihood of reporting motivation to work. For the following stress-related items, HCWs with strong responses to the stress-related item had increased odds of reporting motivation to work at study initiation: “I feel protected by the state and local government” (Up = 0.014, multivariate (M) *p* = 0.007) and “I feel I am protected by my hospital” (Up = 0.009, Mp = 0.008). For the following stress-related items, HCWs with strong responses to the stress-related item had decreased odds of reporting motivation to work: “I feel the quality of my work has decreased” (Up < 0.001, Mp = 0.001), “I feel people are avoiding me” (Mp < 0.001), “I feel anxious about compensation in case I am infected” (Up = 0.044), “I have insomnia” (Up < 0.001, Mp = 0.002), “I feel exhausted physically” (Up = 0.007, Mp = 0.02), and “I feel I have no choice and am obligated to work” (Up = 0.035, Mp = 0.024).

**Figure 6:**
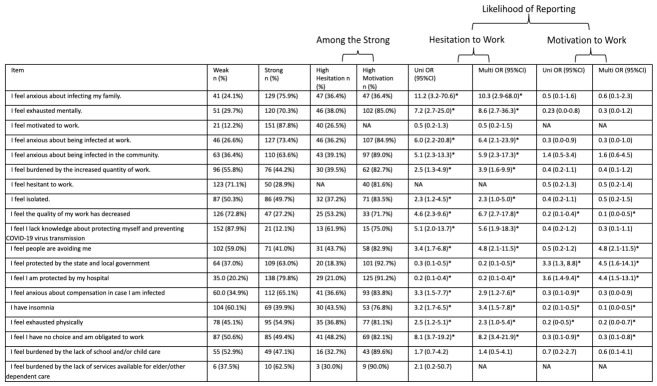
Association of stress factors and likelihood of reporting hesitation and motivation to work at study initiation. Abbreviations: Uni—univariate. Multi—multivariate logistic regression models adjusted for age, gender, job and work location. OR—odds ratio. Notes: * Denotes statistically significant value. NA—Odds ratio values not reported due to small sample size or extreme values. Total of 173 respondents; not all respondents submitted responses to all items.

**Figure 7:**
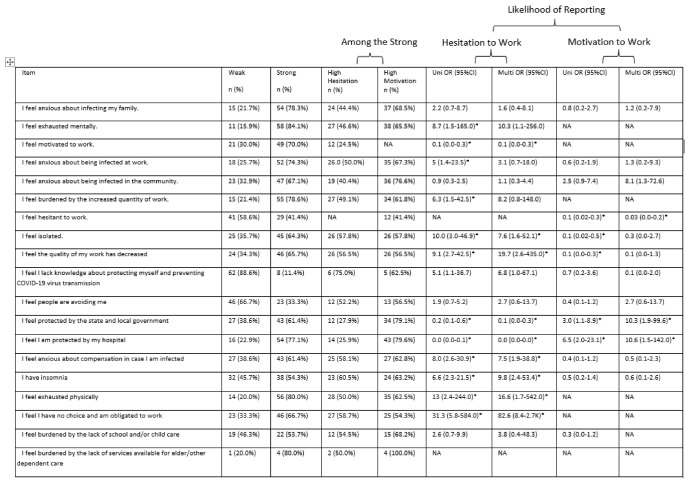
Associations of stress factors and likelihood of reporting hesitation and motivation to work at peak ICU census of COVID-19 patients. Multivariate logistic regression models adjusted for age, gender, job and work location. Abbreviations: Uni—univariate. Multi—multivariate logistic regression models adjusted for age, gender, job and work location. OR—odds ratio. Notes: * Denotes statistically significant value. NA—Odds ratio values not reported due to small sample size or extreme values. Total of 70 respondents; not all respondents submitted responses to all items.

Similarly, at peak ICU census of COVID-19 patients, strong responses to multiple stress-related items were associated with a greater likelihood of reporting motivation to work. For the following stress-related items, HCWs with strong responses to the stress-related item had increased odds of reporting motivation to work at peak ICU census: “I feel protected by the state and local government” (Up = 0.04, multivariate (M) *p* = 0.017) and “I feel I am protected by my hospital” (Up = 0.002, Mp = 0.036). For the following stress-related items, HCWs with strong responses to the stress-related item had decreased odds of reporting motivation to work: “I feel hesitant to work” (Up < 0.001, Mp = 0.005) and “I feel the quality of my work has decreased” (Up = 0.007). The associations of stress factors and the likelihood of reporting motivation to work are shown in Figure 6 and Figure 7.

#### 3.2.3. Hesitation to Work: Demographics

At study initiation, hesitation to work was “high” in 50 (29%) of respondents. This was subdivided as “sometimes hesitant” 40 (23%) and “always hesitant” 10 (6%). There were no statistically significant associations between hesitation to work and age, gender identity, job role, or primary work location. Of note, compared with those working in the “medical ICU”, those working in the “operating room/anesthetizing site” had a trend toward association with hesitation to work (Mp = 0.052).

At peak ICU census of COVID-19 patients, hesitation to work was “high” in 29 (41%) of respondents. This was subdivided into “sometimes hesitant” 17 (24%) and “always hesitant” 12 (17%). At peak ICU census, HCWs who identified as male were less likely to report high hesitation to work compared to the HCWs who identified as female (Up = 0.048). HCWs with a primary work location of cardiothoracic ICU were more likely to report high hesitation to work compared to the reference group of HCWs with a primary work location of medical ICU (Mp = 0.05). The percentage of respondents reporting “always hesitant” to work was greater at peak ICU census compared to study initiation (*p* = 0.0057), whereas there was no difference in the percentage of respondents with “high” hesitation (*p* = 0.063). The associations of demographic characteristics and likelihood of reporting hesitation to work are shown in Figure 4 and Figure 5.

#### 3.2.4. Hesitation to Work: Stress-Related Items

At study initiation, strong responses to multiple stress-related items were associated with a greater likelihood of reporting hesitation to work. For the following stress-related items, HCWs with strong responses to the stress-related item had increased odds of reporting hesitation to work: “I feel anxious about infecting my family” (Up = 0.001, Mp = 0.003), “I am exhausted mentally” (Up < 0.001, Mp < 0.001), “I feel anxious about being infected at work” (Up = 0.001, Mp = 0.002), “I feel anxious about being infected in the community” (Up < 0.001, Mp < 0.001), “I feel burdened by the increased quantity of work” (Up = 0.008, Mp = 0.003), “I feel isolated” (Up = 0.018, Mp = 0.043), “I feel the quality of my work has decreased” (Up < 0.001, Mp < 0.001), “I feel I lack knowledge about protecting myself and preventing COVID-19 virus transmission” (Up < 0.001, Mp = 0.002), “I feel people are avoiding me” (Up < 0.001, Mp < 0.001), “I feel anxious about compensation in case I am infected” (Up = 0.004, Mp = 0.026), “I have insomnia” (Up < 0.001, Mp = 0.003), “I feel exhausted physically” (Up = 0.012, Mp = 0.046) and “I feel I have no choice and am obligated to work” (Up < 0.001, Mp < 0.001). For the following stress-related items, HCWs with strong responses to the stress-related item at study initiation had decreased odds of reporting hesitation to work: “I feel protected by the state and local government” (Up < 0.001, Mp < 0.001) and “I feel I am protected by my hospital” (Up < 0.001, Mp < 0.001).

Similarly, at peak ICU census of COVID-19 patients, strong responses to multiple stress-related items were associated with a greater likelihood of reporting hesitation to work. For the following stress-related items, HCWs with strong responses to the stress-related item had increased odds of reporting hesitation to work: “I feel exhausted mentally” (Up = 0.045), “I feel motivated to work” (Up < 0.001, Mp = 0.002), “I feel anxious about being infected at work” (Up = 0.02), “I feel burdened by the increased quantity of work” (Up = 0.023), “I feel isolated” (Up < 0.001, Mp = 0.019), “I feel the quality of my work has decreased” (Up = 0.001, Mp = 0.013), “I feel anxious about compensation in case I am infected” (Up < 0.001, Mp = 0.008), “I have insomnia” (Up < 0.001, Mp = 0.003), “I feel exhausted physically” (Up = 0.017, Mp = 0.039), and “I feel I have no choice and am obligated to work” (Up = 0.001, Mp = 0.001). For the following stress-related items, HCWs with strong responses to the stress-related item had decreased odds of reporting hesitation to work: “I feel protected by the state and local government” (Up = 0.005, Mp = 0.002) and “I feel I am protected by my hospital” (Up < 0.001, Mp = 0.001). The associations of stress factors and likelihood of reporting hesitation to work are shown in Figure 6 and Figure 7.

#### 3.2.5. WRSS Temporal Relationships

The responses to the WRSS also demonstrated notable trends over time for several of the items. The items “I feel exhausted physically”, “I feel exhausted mentally”, and “I feel hesitant to work” had the greatest percentage of strong responses at peak ICU census, with each having a noticeable reduction post-peak (Supplements S7–S9). The item “I feel motivated to work” had strong responses from 85 to 90% of respondents early in the study, with an acute downtrend at peak ICU census and a rebound post-peak (Supplement S10). The item “I feel anxious about being infected at work” had high (73%) strong responses at study initiation, lower response rates before peak, maximal response rates peri-peak ICU census, and a dramatic reduction post-peak (Supplement S11).

### 3.3. Summary of WRSS Significant Results

Motivation to work and hesitation to work were significantly associated with many demographic and stress-related factors, with changing relationships between pre-peak and peak assessments (summarized in Table 2).

## 4. Discussion

### 4.1. Summary of Findings

This is the first repeated cross-sectional survey to evaluate psychosocial stress before, during and after the first peak of the COVID-19 pandemic in a multidisciplinary sample of ICU HCWs. Our study shows that ICU HCWs had consistently high levels of compassion satisfaction, burnout, and secondary traumatic stress before, during, 3 months following and 6 months following the first peak of COVID-19. High motivation to work was reported by 88% of respondents at study initiation and fell to 70% at peak. High hesitation to work was reported by 29% of respondents at study initiation and increased to 41% at peak.

### 4.2. Placing Findings in the Context of Prior Research and Theoretical Frameworks

First, this study demonstrates a preponderance of ICU HCWs with consistently high CS scores (74–83% of respondents) across all sample points in the study. By comparison, other COVID-19 studies using ProQOL demonstrated substantially lower CS scores. High CS scores were found in 41% of HCWs near the peak and in 33% of HCWs after the first wave [97,114]. Similarly, moderate-to-high-level mean CS scores were found in another sample of ICU HCWs during the pandemic [96]. Because we used scoring methods directly from the ProQOL manual, spurious accounting or faulty analysis is unlikely. Sample-specific characteristics and response bias cannot be ruled out as elaborated below. Our study was not designed to explore why participants selected specific ProQOL responses, and thus we have no quantifiable evidence to explain the high CS scores. Because CS is associated with feelings of fulfillment, reward, achievement, happiness, enrichment, inspiration, energy, gratitude, and hope, we can hypothesize that one or more of these variables was steadily maintained to prevent a substantial reduction in CS across the study period. The study was designed to evaluate temporal trends, and in this regard, CS remained remarkably elevated regardless of changes in the ICU COVID-19 patient census. This suggests that the ICU HCWs continually derived pleasure from the care they provided irrespective of the stress related to the COVID-19 patient burden.

Next, BO has been studied extensively using a variety of instruments, with most meta-analyses reporting 47% to 54% prevalence among HCWs during COVID-19 [22,115,116]. In ICU HCWs specifically, BO has been demonstrated as pervasive and either persistent or worsening throughout the COVID-19 pandemic [7,8,40,42,43,44,46]. Our study found consistently higher levels of BO (74–83% of respondents) across all sample points than typically reported in the literature. Only a few other studies found BO rates around 70% [117,118,119]. Sample-specific characteristics and response bias cannot be ruled out as explanations for persistently elevated BO. Because we used scoring methods directly from the ProQOL manual, spurious accounting or faulty analysis is very unlikely. However, the ProQOL manual does note that the use of cut scores (high/medium/low) is potentially overinclusive, suggesting a risk of a type I error even when following the published scoring instructions [53]. It is possible that our assessment of BO using ProQOL with cut scores identified more individuals with high BO compared to other assessment tools.

Interestingly, in our study, BO was highest at pre-peak measurements (83%) and less at all future time points. In this regard, our findings do not strictly align with the general adaptation syndrome, allostatic overload concept, moral injury framework, and resource depletion model, which hold that with sustained stress, the response changes from resistance to exhaustion with simultaneous increases in BO [49,63,66,73,74,75]. In our study, it is unclear if the stable BO levels reflect a normalization with adaptive stress response. Importantly, there is no clear evidence that HCWs had a normalization response to the chronic stress of COVID-19. Nevertheless, our hospital system frequently used the term “the new normal” when discussing chronic changes in response to COVID-19, potentially inducing a culture of normalization that is consistent with the findings of stable BO. The slightly decreasing BO of ICU HCWs in our study suggests that the higher workload of peak ICU census was offset by ameliorating factors such as adaptive emotion regulation or psychological flexibility.

This study also found high secondary traumatic stress at all time points (70–81% of respondents), with a steady downtrend across the study. Direct ProQOL-based comparisons to the literature are limited, as other studies incorporated STS as a subscale of CF when reporting their findings [96,97,98,99]. CF is composed of BO and STS, and the subjects in our study had high levels of both BO and STS, equating to elevated CF. In this way, the findings in our study are consistent with those of other studies using ProQOL and reporting CF. Furthermore, STS is marked by fear and work-related trauma, factors assessed by other instruments and consistently reported as high in HCWs in the COVID-19 literature [16,17,18,19,20,21,22,23,24,25,26,27,28,41]. The elevated levels of STS in our study are thus consistent with the increased fear and trauma reported elsewhere during COVID-19. The ICU HCWs in our study had slowly declining STS throughout the study period, indicating they continually experienced traumatic stress while successfully avoiding increases or rapid accumulation of additional STS in the setting of rising COVID-19 ICU census and beyond. This suggests an element of protection from moral injury, resource depletion, and/or emotion dysregulation, as each of these entities has been associated with increased STS [49,73,74,75].

A few consistent relationships among ProQOL subscales were noted. BO and STS were moderately or strongly positively associated across all time points, in agreement with previously published results [120]. Values for both measures declined throughout the study. BO and STS are the two elements of compassion fatigue within the professional quality of life framework. Their consistent correlation indicates that the HCWs were experiencing similar changes in BO and STS throughout the study, and the downtrending rate for both throughout the study suggests that our sample population experienced a reduction in compassion fatigue over time. Furthermore, as discussed previously, CS has been shown to be protective from BO. Our data show a weak and moderately negative association between CS and BO across time points, suggesting agreement with the direction of the relationship in the literature, but the strength of the relationship in our study is not strong enough to claim that CS protected our subjects from BO.

Furthermore, this is the only study we are aware of to investigate willingness to work across serial time points during a pandemic. Our study showed that the percentage of HCWs reporting high motivation to work underwent a statistically significant decline from study initiation to peak. At the same time, high hesitation to work had a non-significant increase from study initiation to peak; however, there was a notable increase in HCWs reporting “always hesitant” to work from study initiation to peak. Although factors that decrease motivation do not necessarily increase hesitation, the trend in our study showed a decline in motivation to work and an increase in hesitation to work from pre-peak to peak ICU census. Perhaps this trend is expected given the increasingly stressful working conditions during the rise of the COVID-19 pandemic. The dropping motivation to work and rising hesitation to work from pre-peak to peak COVID-19 ICU census imply that shifting conditions were associated with changes in willingness to work but provide only a global trend without revealing the underlying factors by themselves.

Notably, at study initiation, high motivation to work was not associated with hesitation and high hesitation to work was not associated with motivation; however, at ICU peak census, HCWs reporting high motivation reported low hesitation, and HCWs reporting high hesitation reported low motivation. Thus, it appears that while motivation to work decreased and hesitation to work increased in general for the entire study population from study initiation to peak, there was a subgroup of HCWs with a strong exception to this trend in that they were both highly motivated and failed to experience increased hesitation at the peak. Identifying the existence of this unique group of HCWs is valuable as it demonstrates that it is possible to remain highly motivated and avoid hesitation to work during a pandemic when the trend of most ICU HCWs is just the opposite. Compared with other stress models that explain why and how stress response changes over time for people in general, the frameworks of emotion regulation and psychological flexibility specifically aim to elucidate differences in *individual* stress responses [78,79,80]. This sustained motivation in the face of increasingly stressful work conditions may be reflective of psychological flexibility and/or emotion regulation given its durability over time. Prior work has demonstrated that increased psychological flexibility corresponded with increased work engagement and partially mediated the effects of job satisfaction on mental well-being [58,74]. Future work to identify and understand the mental strategies of highly motivated HCWs during difficult times has potential value for further informing the constructs of emotion regulation and psychological flexibility.

Research surrounding the severe acute respiratory syndrome (SARS) crisis, avian influenza, and H1N1 influenza pandemic indicated that 20 to 30% of HCWs were hesitant or unwilling to work during an infectious pandemic [103,104,121,122,123]. The hesitation of 20–30% of HCWs in these studies is roughly equivalent to the 29–41% of hesitant ICU HCWs in our study, but differing methodologies prevent detailed comparison to our data. However, the best direct comparison available in the literature comes from a survey performed shortly after the 2009 H1N1 pandemic, where Imai et al. found 28.4% of HCWs were “always motivated to work” and 14.7% were “always hesitant to work” [62]. The same 4-point Likert scale questions in our study resulted in “always motivated to work” responses of 35% pre-peak and 26% at peak. Thus, our study population had more HCWs always motivated to work pre-peak but similar responses at COVID-19 peak when compared to HCWs reflecting on the 2009 H1N1 pandemic. At the same time, our study resulted in “always hesitant to work” responses of 6% pre-peak and 17% at peak. Here our study population was less hesitant to work pre-peak but similarly hesitant to work at peak when compared to HCWs reflecting on the 2009 H1N1 pandemic. We can infer from these data that similar proportions of ICU HCWs experienced extreme (“always”) motivation and hesitation to work at peak COVID-19 ICU census when compared to HCWs during the 2009 H1N1 pandemic.

Our data demonstrated that factors of age, gender identity, job role, and primary work location were not associated with either motivation to work or hesitation to work before the first peak of COVID-19; however, at the peak, females had more hesitation to work compared to males. Our data are consistent with prior data showing female HCWs with more hesitation to work than male HCWs during the 2009 H1N1 [62]. A large body of data indicates that female HCWs experienced significantly more psychological sequelae during the COVID-19 pandemic than male HCWs [7,16,17,18,19,20,21,22,23,24,25,26,27,41]. Additional factors obfuscate the role of female gender identity per se, with alternative explanations such as child/elder care responsibilities, occupational role distribution (especially female representation among nurses), and differential exposure to stressors as possible associated factors. Given the complex relationship between female gender, work stress, and the psychosocial response to work stress, we are unable to hypothesize the reasons underlying our finding of female HCWs experiencing greater hesitation to work than male HCWs at peak ICU census.

Years of work experience among a sample of ICU nurses moderated a direct link between emotion regulation difficulties and perceived stress such that greater difficulties in emotion regulation were associated with higher levels of perceived stress in younger nurses [79]. While perceived stress is not the same as motivation or hesitation to work, higher levels of perceived stress are proxies for burnout and declining enthusiasm for work. Our study demonstrated no relationship between multidisciplinary ICU HCW age and willingness to work. Our study also showed that job role was unrelated to willingness to work, suggesting that the role of nursing did not appreciably influence willingness to work when compared to other job roles.

Moreover, our data show HCWs in the cardiothoracic ICU had increased hesitation to work compared to HCWs in other work locations. The reasons for this relationship are unclear, as all ICUs had COVID-19 patients, and the medical ICU had the largest burden by far with dedicated COVID-19 sections. Perhaps this relationship is not based entirely upon COVID-19 factors but also reflects poorly understood nuances of the social dynamics, local culture, workload, and organizational structure of different ICUs [124,125,126,127,128,129,130]. Simply knowing that hesitation to work differs between locations *within the same hospital* suggests a role for customizing stress mitigation interventions for individual units even within the same hospital system. Future work in this area should aim to elucidate location-specific factors that could be leveraged to optimize unit-level stress mitigation beyond institutional-level, broadly applied wellness strategies.

Some HCWs provided care in both the operating room/anesthetizing sites and the ICU during the pandemic. This sub-population included HCWs who worked in both locations pre-pandemic, such as surgeons and anesthesiologists with critical care board certification and nurses who staffed both ICUs and perioperative areas. Other HCWs who previously worked solely in the operating room/anesthetizing sites were reassigned to intermittent ICU duties during portions of the pandemic. Our hospital called for non-intensivist anesthesiologists and operating room nurses to work in the ICUs to help manage the high demand created by the pandemic. Anesthetizing site/OR was included as a survey work location to allow staff with multiple roles to easily identify their primary work location for the 2 weeks preceding a survey. These demographic divisions assured us that we captured the roles and work locations as accurately as possible. We found that ICU HCWs allocated to the operating room/anesthetizing sites had significantly increased motivation to work compared to HCWs in ICU locations. From this interesting relationship, we can hypothesize that differing location-dependent stressors contributed to the discrepancy in motivation to work between operating room/anesthetizing sites and ICUs. For example, it is possible that the acuity burden was diminished in operating room/anesthetizing sites compared to ICUs during the pandemic, allowing HCWs with multiple work sites an opportunity to reinvigorate their motivation to work when not in the ICU. That HCWs showed differing motivation across work locations points to a need to better understand the desirable traits in favorable work locations in order to properly contextualize work locations with less motivated HCWs. Prior work indicates that effective interprofessional collaboration, well-designed facilities, manageable workloads, and supportive organizational culture contribute to a desirable medical workplace [127,129,131,132,133,134,135]. Such nuanced relationships deserve further exploration to allow better adaptation to the stressful ICU environment during routine work as well as future pandemics.

Additionally, motivation to work and hesitation to work were significantly associated with many stress-related factors (Table 2). Concerns about becoming infected, feelings of isolation, and exhaustion were associated with high hesitation to work at study initiation and peak. These findings generally agree with prior work [62]. Within the context of the emotion regulation framework, higher perceived stress and threat cognitive appraisal are associated with lower motivation to work [58,83]. Our results appear to fall in line with this framework in that HCWs hesitant to work had heightened concerns about becoming infected, feelings of isolation, and exhaustion—all perceptions of stress and threat. Furthermore, experiential avoidance was associated with high psychological impact in a prior study [92]. The “feelings of isolation” identified in our study may result from deliberate experiential avoidance in times of stress. Specifically, psychological inflexibility, as characterized by experiential avoidance and emotion-driven behaviors, could manifest as a feeling of isolation as the stressed HCW seeks isolation [79,136]. Furthermore, rising physical and mental exhaustion with apex at peak ICU census of COVID-19 patients (see Supplements S7 and S8) suggests increasing exhaustion with ongoing and increasing stress, as predicted by the general adaptation syndrome framework and the allostatic load model [63,64,65,66]. Exhaustion levels did drop after peak census, suggesting an element of recovery following the peak.

Feeling protected by the government and hospital was associated with decreased hesitation and increased motivation, suggesting that awareness of large-scale interventions to protect HCWs is an important element of maintaining the workforce during a pandemic. This finding aligns with the Job Demands-Resources model, wherein organizational support reduces HCW stress by buffering the negative effects of job demands [137]. Similarly, because “feeling protected by the hospital” is a feeling and not necessarily an objective fact, our finding that feeling protected by the hospital was associated with decreased hesitation and increased motivation fits well within the framework of the Perceived Organizational Support theory, wherein an employee’s beliefs about their institution’s concern for their well-being affects their stress level [138]. Feelings about hospital and government protection also reflect HCW perspectives about safety equipment, safety procedures, and vaccinations. From this perspective, the availability of resources translates to lower moral injury and a greater conservation of resources for coping, thus conforming to the construct of moral injury and the conservation of resources theory.

HCW concerns about infecting family were associated with hesitation to work at study initiation only. HCWs who felt they had no choice and were obligated to work had higher hesitation at study initiation and peak. These responses reflect an association between moral injury and hesitation to work in that they potentiate an imbalance between patient care obligations and HCW concern for self and family safety [69,70,71].

Concerns regarding the lack of childcare and the lack of elder care services did not affect willingness to work. It seems that, to some extent, these variables were unimportant for HCW willingness to work within our sample, possibly from compartmentalization of home and work life or by availability of sufficient resources.

### 4.3. Practical Implications

Taken together, a few cautious implications can be derived from this work. We demonstrated that HCWs can experience high levels of CS and BO simultaneously, suggesting that interventions for HCW well-being could focus on both reducing BO and increasing CS. Considerations for comprehensive interventions would aim at fostering feelings of fulfillment, reward, achievement, happiness, enrichment, inspiration, energy, and gratitude, with accompanying BO reduction strategies of limiting exhaustion, frustration, anger and depression. Next, our findings emphasize that motivation to work is not necessarily the opposite of hesitation to work. This implies that optimal motivation strategies during crises may work to boost motivation and reduce hesitation as two separate mechanistic strategies. The identification of a group of HCWs with high motivation and low hesitation at peak census despite exactly the opposite of these trends in the sample as a whole suggests a distinct role for individual responses to stress (rather than global, similar responses among HCWs). Finding a similar group of people in future studies and elucidating their coping mechanisms provides an opportunity to inform existing theoretical frameworks and carry over to practice. ICU subtype (site-specific) differences in willingness to work point to an important role of localized culture within a hospital system. Identifying whether hospital-wide stress mitigation interventions are optimal compared to locally customized interventions is a consideration for future research. Finally, HCW feelings about protection by the government and hospital highlight the important role of organization-level culture and interventions, implying that system-wide support continues to have a role in HCW wellness during a crisis.

### 4.4. Strengths and Limitations

Our study has several strengths. First, the ProQOL is well-suited for this purpose as it is intended expressly for health care professionals as a research measure of the positive and negative effects of working with people who have experienced extremely stressful events. Similarly, the WRSS was initially designed for the evaluation of willingness to work during the 2009 H1N1 pandemic and thus has specific applicability to studying pandemic conditions. Also, our study uses a repeated cross-sectional method, significantly less commonly used in COVID-19. Prior research on the psychological impact of the 2003 SARS epidemic, the 2009 H1N1 pandemic, the 2016 Middle East respiratory syndrome (MERS) epidemic, and the 2014–2016 West Africa Ebola epidemic showed that most cross-sectional studies evaluated a single time point [139]. We sought to improve on prior infectious outbreak research by employing an assessment at multiple time points. At the time of study initiation, our institution had maintained a relatively consistent low inpatient census from the onset of the pandemic. This afforded us the opportunity to assess psychosocial factors in ICU professionals before, during, and after the first regional COVID-19 peak. Additionally, we surveyed our study population in real time and frequently during the pandemic. This technique allowed for comparisons of specific time points and did not rely on respondents’ recollection of events greater than 2 weeks past.

Our assessments within a single institution mean that all HCWs experienced the same hospital-wide protocols for safety, wellness, equipment, etc. Over the study period there were numerous changes that potentially affected ICU HCWs’ wellness, such as a transition from a shortage of personal protection equipment to ubiquitous availability, initiation of COVID-19 vaccinations, and improvements in viral testing techniques. While we cannot control for the effects of these changes, all ICU HCWs in our study experienced these changes simultaneously. The sample population was homogeneous in this way, making chronological comparisons reflective of the queried factors and unaffected by regional differences that affect multi-institutional or review studies. Moreover, our tertiary referral center’s model of multiple specialty-specific ICUs is common among larger hospitals, making our findings generalizable to other health care systems with a similar model.

Our study has several weaknesses. The validity of the ProQOL has been questioned, and research suggests that CS and CF represent upper and lower levels of the same construct rather than distinct constructs [140]. However, previous testing indicated acceptable levels of internal consistency and reliability for each of the subscales; the Cronbach alpha was 0.88 for CS, 0.75 for BO, and 0.81 for STS [141]. This is further supported by a study on HCWs during the COVID-19 pandemic, where Cronbach’s alpha was 0.87 for CS, 0.70 for BO, and 0.84 for STS [114].

The dichotomization of ordinal WRSS responses (never, rarely, sometimes, always) into weak/strong categories potentially resulted in loss of information. Dichotomization was chosen both for simplicity of comparisons and to reflect the practical emotional states of either being motivated or not and being hesitant or not, rather than trying to assess with multiple degrees of willingness to work. Also, because motivation and hesitation to work were used for logistic regression models at multiple time points against 17 WRSS items, analysis incorporating multiple degrees of willingness to work may have become statistically problematic with dilution of sample sizes into multiple subgroups in the context of an already diminishing sample size for the study overall. The differential display of “sometimes” and “always” responses in Supplemental Figures preserves the fidelity of some of this information when viewed graphically over time.

Additionally, while other studies have measured factors similar to CS and STS, direct comparisons are limited to the few studies that used ProQOL as the assessment tool. Similarly, BO has been directly assessed by many instruments in ICU HCWs, but inter-study comparisons are limited to general trends when differing instruments have been employed.

Another weakness is declining survey response rates. The ICU HCWs in this study likely had survey fatigue given the frequency of study surveys in the context of institutional daily COVID screening surveys and weekly wellness surveys. Declining response rates (from 33% to 10% for ProQOL; 27% to 5.7% for WRSS) substantially increase the risk of non-response bias. With diminishing survey respondents, the 3-month post-peak WRSS subgroup sample sizes were too small for meaningful comparisons.

Gradual changes in staff location, job type, and employment status contributed to small shifts in the study population. Later respondents may have differed systematically from earlier respondents. If respondents to later surveys differed systematically from those of earlier surveys, such specific attrition would cause temporal changes in metrics to be distorted rather than demonstrating genuine trends in the study population. For example, sample-wide changes in emotional regulation or psychological flexibility over the course of the study would introduce error to our measurements. We did not control for changes in survey recipients or which recipients chose to respond. Because we compared aggregate responses between time points, we were unable to evaluate changes in each individual respondent over time. Overall, there is a risk that the data reflect, in part, changes in who was queried and who responded (non-response bias) and systematic changes in the sample population over time. There is also the possibility of selective attrition (e.g., those with highest stress levels may have responded with disengagement, and disengagement could include choosing to not engage in the study and other surveys). Anecdotally, there were no dramatic changes in ICU staffing aside from job type reassignment, which we attempted to account for by asking the primary job type in the 2 weeks preceding survey completion.

## 5. Conclusions

This is the first repeated cross-sectional survey to evaluate the psychosocial effects of the COVID-19 pandemic in multidisciplinary ICU HCWs before, during, and after the first peak. In summary, ICU HCWs reported high levels of stress at all measured time points and experienced unfavorable changes in willingness to work at peak ICU census. Motivation to work declined, and hesitation to work increased from pre-peak to peak conditions. Motivation to work was associated with ICU HCWs feeling protected by the government and hospital. Hesitation to work was associated with ICU HCWs’ concern about becoming infected, feelings of isolation, and exhaustion. Willingness to work varied across work locations. Because study methodology precludes causal inference and low survey response rates indicate that findings should be interpreted with caution, these results are best viewed as hypothesis-generating for future work aimed at improving stress mitigation in ICU HCWs.

## Figures and Tables

**Table 1:** Professional Quality Of Life Measure (ProQOL) results.

	Initial	Peak	3 Months Post-Peak	6 Months Post-Peak
Number of Respondents n (%)	205 (33%)	92 (16%)	64 (11%)	61 (10%)
**Compassion Satisfaction**				
High	166 (81%)	72 (78%)	53 (83%)	45 (74%)
Moderate	37 (18%)	20 (22%)	9 (14%)	13 (21%)
Low	2 (1%)	-	2 (3%)	3 (5%)
**Burnout**				
High	170 (83%)	68 (74%)	51 (80%)	48 (79%)
Moderate	35 (17%)	24 (26%)	13 (20%)	13 (21%)
Low	-	-	-	-
**Secondary Traumatic Stress**				
High	166 (81%)	69 (75%)	46 (72%)	43 (70%)
Moderate	39 (19%)	23 (25%)	18 (28%)	18 (30%)
Low	-	-	-	-

**Table 2:** Summary of Work-Related Stress Survey: statistically significant associations between demographic/stress-related factors and willingness to work at study initiation and peak ICU census.

WRSS Item	Association(s) at Study Initiation	Association(s) at Peak ICU Census
Reported high motivation to work	88%	70%
Reported high hesitation to work	29%	41%
Age	none	none
Gender identity	none	females more hesitation
Job role	none	none
Primary work location	none	cardiothoracic ICU more hesitation, OR/anesth more motivation
I feel anxious about infecting my family.	increased hesitation	none
I feel exhausted mentally.	increased hesitation	increased hesitation
I feel motivated to work.	none	decreased hesitation
I feel anxious about being infected at work.	increased hesitation	increased hesitation
I feel anxious about being infected in the community.	increased hesitation	none
I feel burdened by the increased quantity of work.	increased hesitation	increased hesitation
I feel hesitant to work.	none	decreased motivation
I feel isolated.	increased hesitation	increased hesitation
I feel the quality of my work has decreased	increased hesitation, decreased motivation	increased hesitation, decreased motivation
I feel I lack knowledge about protecting myself and preventing COVID-19 virus transmission	increased hesitation	increased hesitation
I feel people are avoiding me	increased hesitation, increased motivation	none
I feel protected by the state and local government	decreased hesitation, increased motivation	decreased hesitation, increased motivation
I feel I am protected by my hospital	decreased hesitation, increased motivation	decreased hesitation, increased motivation
I feel anxious about compensation in case I am infected	increased hesitation, decreased motivation	increased hesitation
I have insomnia	increased hesitation, decreased motivation	increased hesitation
I feel exhausted physically	increased hesitation, decreased motivation	increased hesitation
I feel I have no choice and am obligated to work	increased hesitation, decreased motivation	increased hesitation
I feel burdened by the lack of school and/or child care	none	none
I feel burdened by the lack of services available for elder/other dependent care	none	none

## Data Availability

The raw data supporting the conclusions of this article are restricted due to privacy concerns. The granularity of the raw data potentially allows private information (e.g., income, gender identity) to be linked to specific individuals through the process of elimination by other identifiers (e.g., age, job role, work location). Upon request, the corresponding author will petition the local internal review board for permission to share raw data for research purposes.

## Supplementary Materials

The following supporting information can be downloaded at: https://www.mdpi.com/article/10.3390/healthcare14091154/s1, S1: Checklist for Reporting of Survey Studies (CROSS); S2: COVID-19 ProQOL Survey; S3: COVID-19 WRSS; S4: ProQOL aggregate respondent characteristics; S5: WRSS response rates; S6: WRSS aggregate respondent characteristics; S7: WRSS strong responses to “I am physically exhausted”; S8: WRSS strong responses to “I am mentally exhausted”; S9: WRSS strong responses to “I feel hesitant to work”; S10: WRSS strong responses to “I am motivated to work”; S11: WRSS strong responses to “I feel anxious about being infected at work”; S12: WRSS free-text responses word clouds.

## Abbreviations

The following abbreviations are used in this manuscript:

COVID-19	Coronavirus disease 2019
ICU	Intensive care unit
HCW	Health care worker
BO	Burnout
CS	Compassion satisfaction
STS	Secondary traumatic stress
ProQOL	Professional Quality of Life Measure Version 5
WRSS	Work-Related Stress Survey
APP	Advanced practice provider
Up	Univariate *p*-value
