# Supplementary material for: Psychosocial Impact of COVID-19 on Intensive Care Unit Personnel: A Repeated Cross-Sectional Survey Assessment Before, During, and After the First Peak
Source: Healthcare (Basel). 2026 Apr 25;14(9):1154. doi: 10.3390/healthcare14091154 (PMC13163874; doi:10.3390/healthcare14091154)
Supplement: Supplementary file 1 [file healthcare-14-01154-s001.zip › S2.pdf]

Covid-19 PRoQOL Survey

This is a professional quality of life survey.

Your responses to this survey may contain information that could potentially be used to identify you. Response to the survey reflects voluntary participation in the study and no signature is needed for consent.

Please consider the statements below in context of caring for patients in the past two weeks.

Thank you!

Demographics Questions

	18-29	30-39	40-49	50-59	60-69	70+
Age (years)	◯	◯	◯	◯	◯	◯

Gender identity

◯ Male ◯ Female ◯ Non-binary

Job role

◯ Physician
◯ Advance Practice Provider (Nurse Practitioner or Physician Assistant)
◯ Certified Registered Nurse Anesthetist or Anesthesia Assistant
◯ Pharmacist
◯ Nurse
◯ Nursing Assistant/Tech
◯ Other

Physician type

◯ Surgical/medical intensivist
◯ Anesthesiologist critical care fellowship trained
◯ Anesthesiologist non-critical care fellowship trained

Primary work location in the past two weeks

◯ Neuro ICU
◯ Surgical ICU
◯ Cardiothoracic ICU
◯ Medical ICU
◯ Other ICU
◯ Operating room/anesthetizing site

Years of Experience

◯ 0-5 ◯ 6-10 ◯ 11-15
◯ 16-20 ◯ >20

Martial status

◯ Single
◯ Married or in a domestic partnership
◯ Divorced
◯ Widowed
◯ Seperated

Household income (pre-tax totals for all adults in the household for the 2019 calendar year).	◯ < $24,999<br>◯ $25,000-$39,999<br>◯ $40,000-$54,999<br>◯ $55,000-$69,999<br>◯ $70,000-$89,999<br>◯ $90,000-$119,999<br>◯ $120,000-$159,999<br>◯ $160,000-$199,999<br>◯ $200,000-$299,999<br>◯ $300,000-$399,999<br>◯ $400,000-$599,999<br>◯ >$600,000
Employment status	◯ Full Time ◯ Part Time
Self-described health status	◯ Poor ◯ Fair ◯ Good<br>◯ Very Good ◯ Excellent

PRoQOL Survey: Please consider the statements below in context of caring for patients in the past two weeks and that use of the words trauma or traumatic in the survey apply to all patients you are caring for.

		Never	Rarely	Sometimes	Often	Very Often
1	I am happy.	◯	◯	◯	◯	◯
2	I am preoccupied with more than one person I care for.	◯	◯	◯	◯	◯
3	I get satisfaction from being able to care for people.	◯	◯	◯	◯	◯
4	I feel connected to others.	◯	◯	◯	◯	◯
5	I jump or am startled by unexpected sounds.	◯	◯	◯	◯	◯
6	I feel invigorated after working with those I care for.	◯	◯	◯	◯	◯
7	I find it difficult to separate my personal life from my life as a caregiver.	◯	◯	◯	◯	◯
8	I am not as productive at work because I am losing sleep over traumatic experiences of a person I cared for.	◯	◯	◯	◯	◯
9	I think that I might have been affected by the traumatic stress of those I care for.	◯	◯	◯	◯	◯
10	I feel trapped by my job as a caregiver.	◯	◯	◯	◯	◯
11						

	Because of my caregiving, I have felt "on edge" about various things.	○	○	○	○	○
12	I like my work as a caregiver.	○	○	○	○	○
13	I feel depressed because of the traumatic experiences of the people I care for.	○	○	○	○	○
14	I feel as though I am experiencing the trauma of someone I have cared for.	○	○	○	○	○
15	I have beliefs that sustain me.	○	○	○	○	○
16	I am pleased with how I am able to keep up with health care techniques and protocols.	○	○	○	○	○
17	I am the person I always wanted to be.	○	○	○	○	○
18	My work makes me feel satisfied.	○	○	○	○	○
19	I feel worn out because of my work as a caregiver.	○	○	○	○	○
20	I have happy thoughts and feelings about those I care for and how I could help them.	○	○	○	○	○
21	I feel overwhelmed because my case load seems endless.	○	○	○	○	○
22	I believe I can make a difference through my work.	○	○	○	○	○
23	I avoid certain activities or situations because they remind me of frightening experiences of the people I care for.	○	○	○	○	○
24	I am proud of what I can do as a caregiver.	○	○	○	○	○
25	As a result of my caregiving, I have intrusive, frightening thoughts.	○	○	○	○	○
26	I feel "bogged down" by the system.	○	○	○	○	○
27	I have thoughts that I am a "success" as a caregiver.	○	○	○	○	○
28	I can't recall important parts of my work with trauma victims.	○	○	○	○	○
29	I am a very caring person.	○	○	○	○	○
30						

I am happy that I chose to do this work.	○	○	○	○	○

Please identify any stress reduction techniques you have used in the past week

____

Additional comments about your role as a caregiver during the COVID-19 pandemic

____

CS Subscale Score

____

BO Subscale Score

____

STS Subscale Score

____
