# Supplementary material for: Psychosocial Impact of COVID-19 on Intensive Care Unit Personnel: A Repeated Cross-Sectional Survey Assessment Before, During, and After the First Peak
Source: Healthcare (Basel). 2026 Apr 25;14(9):1154. doi: 10.3390/healthcare14091154 (PMC13163874; doi:10.3390/healthcare14091154)
Supplement: Supplementary file 1 [file healthcare-14-01154-s001.zip › S3.pdf]

Covid-19 Work-Related Stress Survey

This is a Work-Related Stress survey for the COVID-19 pandemic.

Your responses to this survey may contain information that could potentially be used to identify you. Response to the survey reflects voluntary participation in the study and no signature is needed for consent.

Please consider the statements below in context of caring for patients in the past week.

Thank you!

Demographics Questions

	18-29	30-39	40-49	50-59	60-69	70+
Age (years)	◯	◯	◯	◯	◯	◯

Gender identity
- ◯ Male
- ◯ Female
- ◯ Non-binary

Job role
- ◯ Physician
- ◯ Advance Practice Provider (Nurse Practitioner or Physician Assistant)
- ◯ Certified Registered Nurse Anesthetist or Anesthesia Assistant
- ◯ Pharmacist
- ◯ Nurse
- ◯ Nursing Assistant/Tech
- ◯ Other

Physician type
- ◯ Surgical/medical intensivist
- ◯ Anesthesiologist critical care fellowship trained
- ◯ Anesthesiologist non-critical care fellowship trained

Primary work location in the past two weeks
- ◯ Neuro ICU
- ◯ Surgical ICU
- ◯ Cardiothoracic ICU
- ◯ Medical ICU
- ◯ Other ICU
- ◯ Operating room/anesthetizing site

	0-5	6-10	11-15	16-20	>20
Years of experience	◯	◯	◯	◯	◯

Martial status
- ◯ Single
- ◯ Married or in a domestic partnership
- ◯ Divorced
- ◯ Widowed
- ◯ Seperated

Household income (pre-tax totals for all adults in the household for the 2019 calendar year).	◯ < $24,999<br>◯ $25,000-$39,999<br>◯ $40,000-$54,999<br>◯ $55,000-$69,999<br>◯ $70,000-$89,999<br>◯ $90,000-$119,999<br>◯ $120,000-$159,999<br>◯ $160,000-$199,999<br>◯ $200,000-$299,999<br>◯ $300,000-$399,999<br>◯ $400,000-$599,999<br>◯ >$600,000
Employment status	◯ Full Time<br>◯ Part Time
Self-described health status	◯ Poor<br>◯ Fair<br>◯ Good<br>◯ Very Good<br>◯ Excellent

Please rate how you have felt during the past week.

		Never	Rarely	Sometimes	Always
1	I feel anxious about being infected at work.	◯	◯	◯	◯
2	I feel anxious about infecting my family.	◯	◯	◯	◯
3	I feel burdened by the increased quantity of work.	◯	◯	◯	◯
4	I feel the quality of my work has decreased.	◯	◯	◯	◯
5	I feel anxious about being infected in the community.	◯	◯	◯	◯
6	I feel I lack knowledge about protecting myself and preventing COVID-19 virus transmission.	◯	◯	◯	◯
7	I feel people are avoiding me in the community	◯	◯	◯	◯
8	I feel protected by the state and local government.	◯	◯	◯	◯
9	I feel protected by my hospital.	◯	◯	◯	◯
10	I feel anxious about compensation if I get infected.	◯	◯	◯	◯
11	I feel anxious about my job security and compensation in general.	◯	◯	◯	◯
12					

		Never	Rarely	Sometimes	Always
	I feel hesitant to work.	◯	◯	◯	◯
13	I feel isolated.	◯	◯	◯	◯
14	I have insomnia.	◯	◯	◯	◯
15	I am exhausted physically.	◯	◯	◯	◯
16	I am exhausted mentally.	◯	◯	◯	◯
17	I am motivated to work.	◯	◯	◯	◯
18	I feel I have no choice and am obligated to work.	◯	◯	◯	◯
19	When reporting to the hospital for work I feel adequately recovered after my time away	◯	◯	◯	◯
20	I am anxious about my family's health.	◯	◯	◯	◯

Do you have children? ◯ Yes ◯ No

		Never	Rarely	Sometimes	Always
21	I feel burdened by a lack of school and/or child care.	◯	◯	◯	◯

Do you provide elder/other dependent care? ◯ Yes ◯ No

		Never	Rarely	Sometimes	Always
22	I feel burdened by a lack of services available for elder/other dependent care.	◯	◯	◯	◯

		Never	Rarely	Sometimes	Always
23	I have limited my exposure to my family/children/dependents.	◯	◯	◯	◯

I have to pay extra, out of pocket expenses for childcare or elder/dependent care services so I can work. ◯ Yes ◯ No

---

Please identify any stress reduction techniques you have used in the past week

____

Additional comments related to stressors from the COVID-19 pandemic and how it has affected you

____
