# Supplementary material for: Psychosocial Impact of COVID-19 on Intensive Care Unit Personnel: A Repeated Cross-Sectional Survey Assessment Before, During, and After the First Peak
Source: Healthcare (Basel). 2026 Apr 25;14(9):1154. doi: 10.3390/healthcare14091154 (PMC13163874; doi:10.3390/healthcare14091154)
Supplement: Supplementary file 1 [file healthcare-14-01154-s001.zip › S4.pdf]

Characteristic						
Age (years)	18-29<br>76 (28%)	30-39<br>110 (40%)	40-49<br>55 (9%)	50-59<br>24 (9%)	>59<br>11 (4%)	
Gender identity	Male<br>93 (33%)	Female<br>185 (67%)	Non-Binary<br>0			
Have children[1]	Yes<br>173 (62%)	No<br>104 (38%)				
Provide elder care[2]	Yes<br>23 (8%)	No<br>254 (92%)				
Marital status	Married[3]<br>210 (76%)	Single<br>60 (23%)	Divorced<br>5 (3%)	Widowed<br>2 (1%)	Separated<br>1 (1%)	
Health status[4]	Excellent<br>51 (18%)	Very good<br>129 (46%)	Good<br>90 (32%)	Fair<br>8 (3%)	Poor<br>0	
Income[5]	<$25,000<br>6 (2%)	$25,000-39,999<br>7 (3%)	$40,000-54,999<br>13 (5%)	$55,000-69,999<br>29 (11%)	$70,000-89,999<br>27 (10%)	$90,000-119,999<br>33 (12%)

Income[5]	$120,000-159,999<br><br>51 (19%)	$160,000-199,999<br><br>31 (11%)	$200,000-299,999<br><br>19 (7%)	$300,000-399,999<br><br>15 (5%)	$400,000-599,999<br><br>38 (14%)	$600,000-999,999<br><br>5 (2%)
Job role	Nurse<br><br>164 (59%)	Physician<br><br>58 (21%)	APP[6]<br><br>26 (9%)	Other[7]<br><br>30 (11%)		
Physician type[8]	Intensivist<br><br>20 (34%)	Anesthesiologist<br><br>38 (66%)				
Work location[9]	OR/Anes<br><br>71 (26%)	CT ICU<br><br>57 (21%)	SICU<br><br>56 (20%)	MICU<br><br>43 (16%)	NeuroICU<br><br>38 (14%)	Other ICU<br><br>11 (4%)

Supplement 4. Professional Quality of Life Measure (ProQOL) aggregate respondent characteristics.

There were 278 unique individual respondents to one or more ProQOL surveys over the course of the study. Notes: 1. Non-adult children who are dependents. 2. Elder dependent care. 3. Includes domestic partnerships. 4. Self-described health status. 5. Income brackets based on United States Internal Revenue Service Tax Code, reported in U.S. Dollars 2020. 6. Advanced practice provider, defined as a nurse practitioner, certified registered nurse anesthetist, anesthesia assistant, or physician assistant. 7. Other job roles included nursing assistant, nursing technician, and pharmacist. 8. Anesthesiologists working as intensivists were counted only once as anesthesiologists. 9. Primary work location in the past two weeks.

Abbreviations: OR/Anes – Operating room or other anesthetizing site. CT ICU – Cardiothoracic intensive care unit. SICU – Surgical intensive care unit. MICU – Medical intensive care unit. NeuroICU – Neurosciences intensive care unit.
