# Supplementary material for: Psychosocial Impact of COVID-19 on Intensive Care Unit Personnel: A Repeated Cross-Sectional Survey Assessment Before, During, and After the First Peak
Source: Healthcare (Basel). 2026 Apr 25;14(9):1154. doi: 10.3390/healthcare14091154 (PMC13163874; doi:10.3390/healthcare14091154)
Supplement: Supplementary file 1 [file healthcare-14-01154-s001.zip › S5.pdf]

	Number of Respondents	Respondents Solicited	Response Percentage
WRSS 1	173	639	27
WRSS 2	139	634	22
WRSS 3	119	630	19
WRSS 4	100	625	16
WRSS 5	88	618	14
WRSS 6	75	591	13
WRSS 7	66	591	11
WRSS 8	79	587	14
WRSS 9	70	580	12
WRSS 10	51	568	9
WRSS 11	46	566	8
WRSS 12	45	562	8
WRSS 13	32	558	6

Supplement 5. Work-related stress survey (WRSS) response rates.
