# Supplementary material for: Psychosocial Impact of COVID-19 on Intensive Care Unit Personnel: A Repeated Cross-Sectional Survey Assessment Before, During, and After the First Peak
Source: Healthcare (Basel). 2026 Apr 25;14(9):1154. doi: 10.3390/healthcare14091154 (PMC13163874; doi:10.3390/healthcare14091154)
Supplement: Supplementary file 1 [file healthcare-14-01154-s001.zip › S6.pdf]

| Characteristic | | | | | | |
|---|---|---|---|---|---|---|
| Age (years) | 18-29<br>74 (27%) | 30-39<br>108 (39%) | 40-49<br>53 (19%) | 50-59<br>23 (8%) | >59<br>20 (7%) | |
| Gender identity | Male<br>93 (33%) | Female<br>184 (66%) | Non-Binary<br>1 (0.4%) | | | |
| Job Role | Nurse<br>135 (49%) | Physician<br>58 (21%) | APP[1]<br>55 (17%) | Nurse Assistant<br>21(8%) | Pharmacist<br>7 (2.5%) | Other<br>2 (0.7%) |
| Work Location[2] | OR/Anes<br>71 (26%) | CT ICU<br>57 (21%) | SICU<br>56 (20%) | MICU<br>43 (16%) | NeuroICU<br>38 (14%) | Other ICU<br>13 (5%) |

Supplement 6. Work related stress survey (WRSS) aggregate respondent characteristics.

There were 278 unique individual respondents to one or more WRSS over the course of the study. Notes: 1. Advanced practice provider, defined as a nurse practitioner, certified registered nurse anesthetist, anesthesia assistant, or physician assistant. 2. Primary work location in the past two weeks.

Abbreviations: OR/Anes- Operating room or other anesthetizing site. CT ICU – Cardiothoracic intensive care unit. SICU – Surgical intensive care unit. MICU – Medical intensive care unit. NeuroICU – Neurosciences intensive care unit.
