# Supplementary figures and images for: Psychosocial Impact of COVID-19 on Intensive Care Unit Personnel: A Repeated Cross-Sectional Survey Assessment Before, During, and After the First Peak
Source: Healthcare (Basel). 2026 Apr 25;14(9):1154. doi: 10.3390/healthcare14091154 (PMC13163874; doi:10.3390/healthcare14091154)

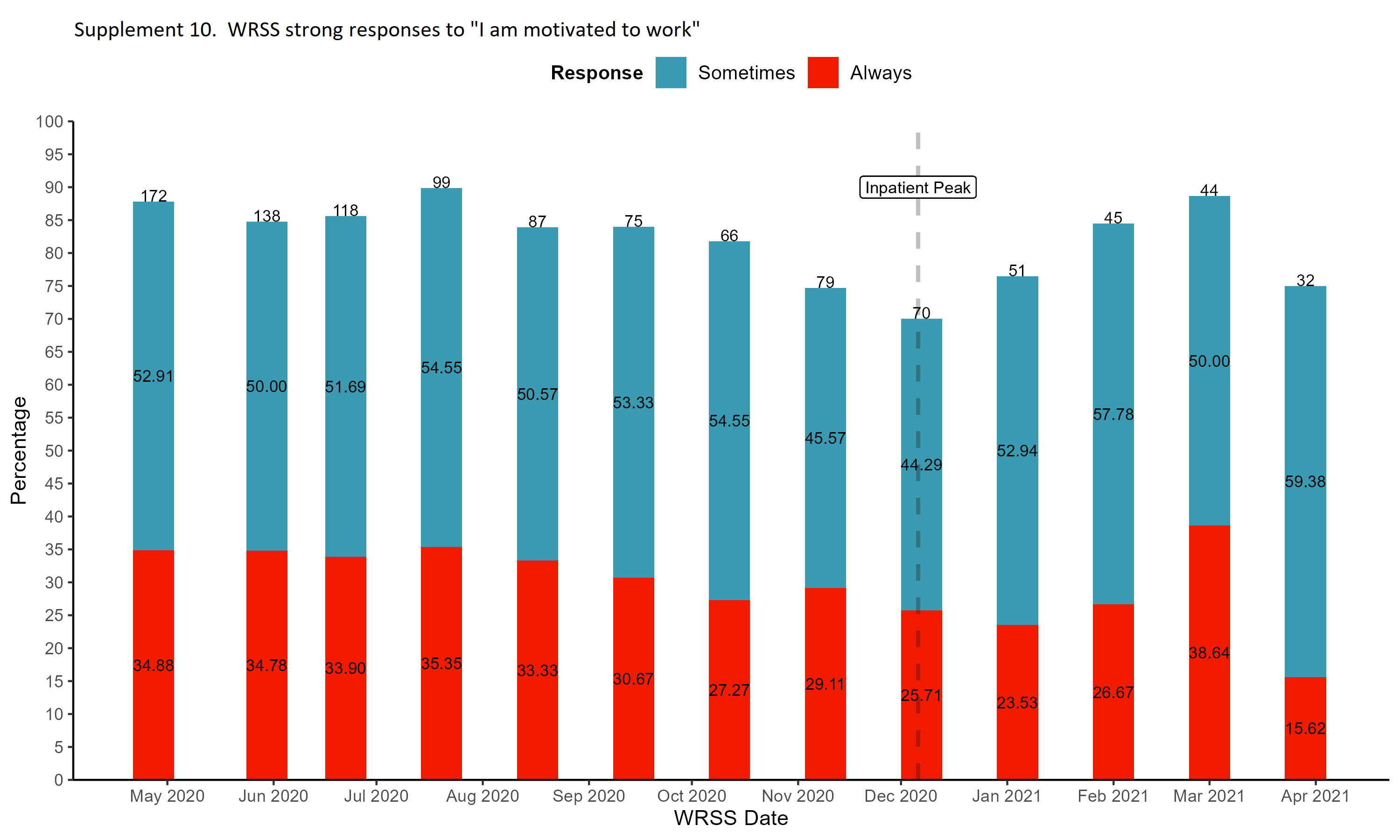

Supplement: Supplementary file 1 [file healthcare-14-01154-s001.zip › S10.tiff]

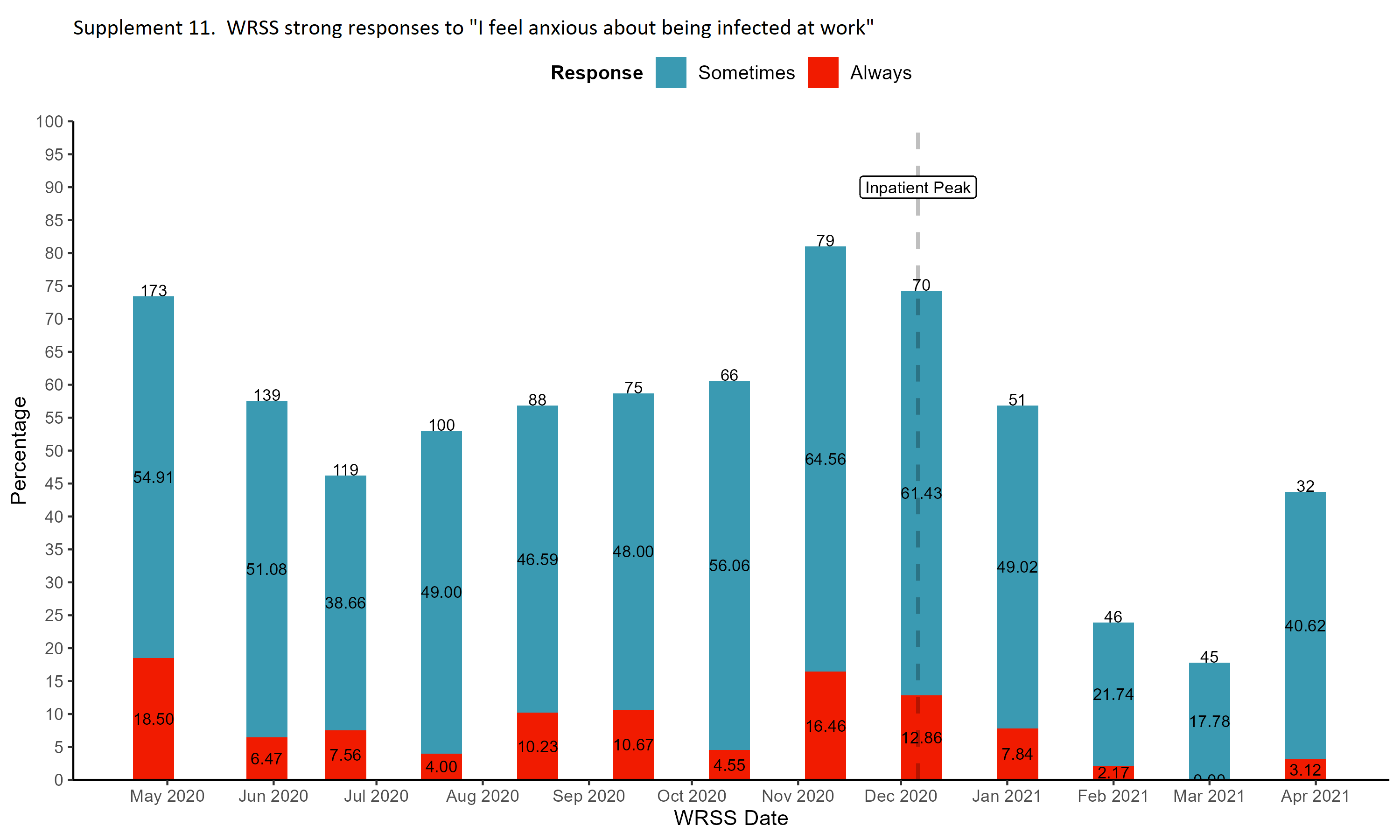

Supplement: Supplementary file 1 [file healthcare-14-01154-s001.zip › S11.tiff]

## Supplement 12. WRSS Free Text Responses Word Clouds

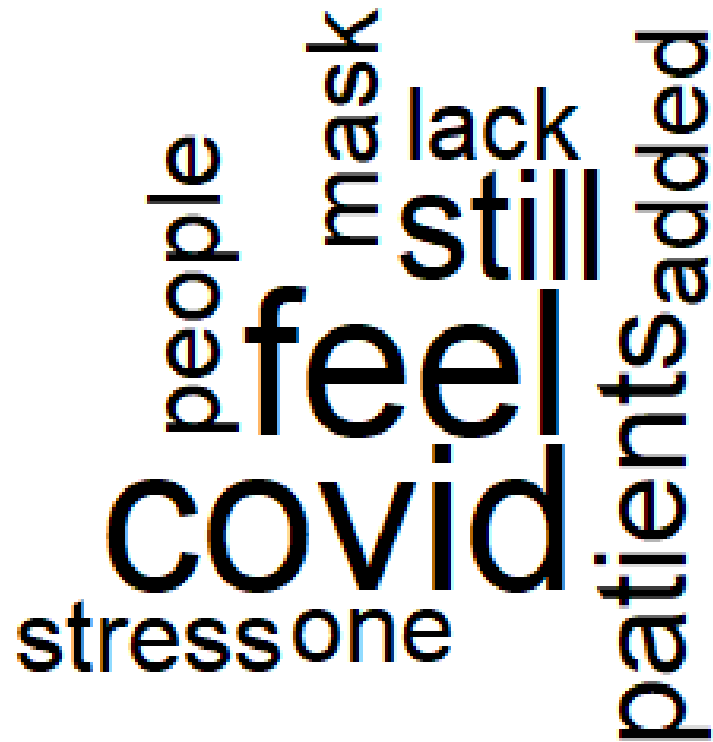

Study Initiation

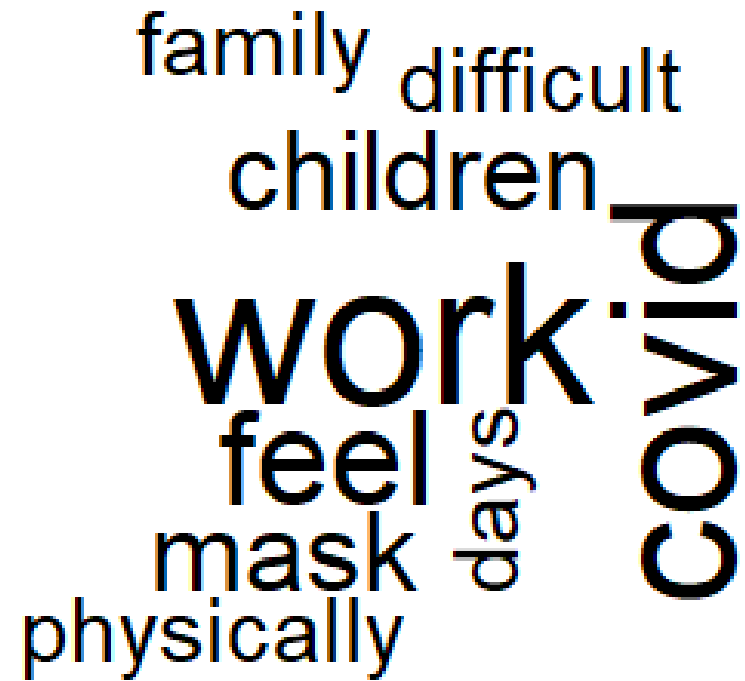

Peak

Supplement: Supplementary file 1 [file healthcare-14-01154-s001.zip › S12.pdf]

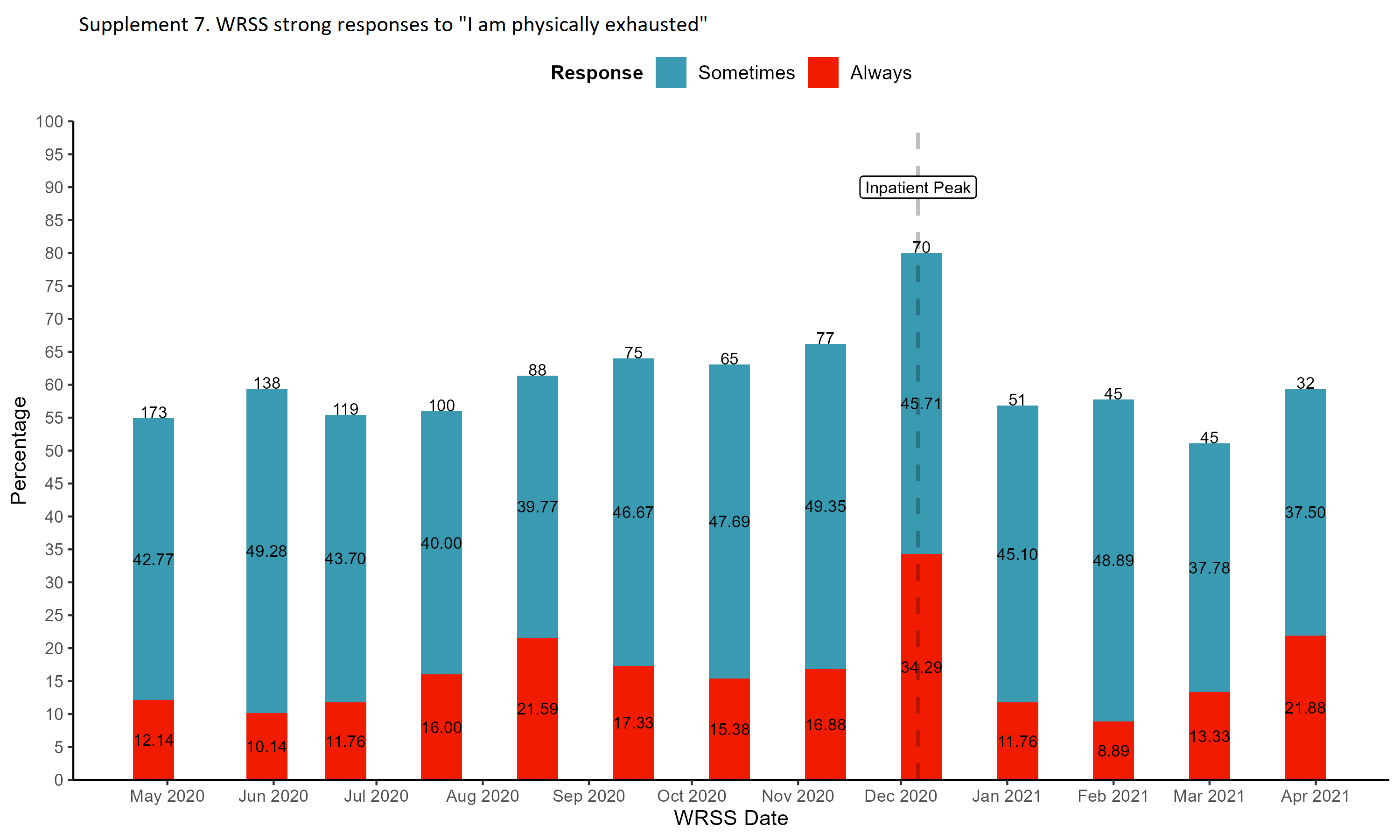

Supplement: Supplementary file 1 [file healthcare-14-01154-s001.zip › S7.tiff]

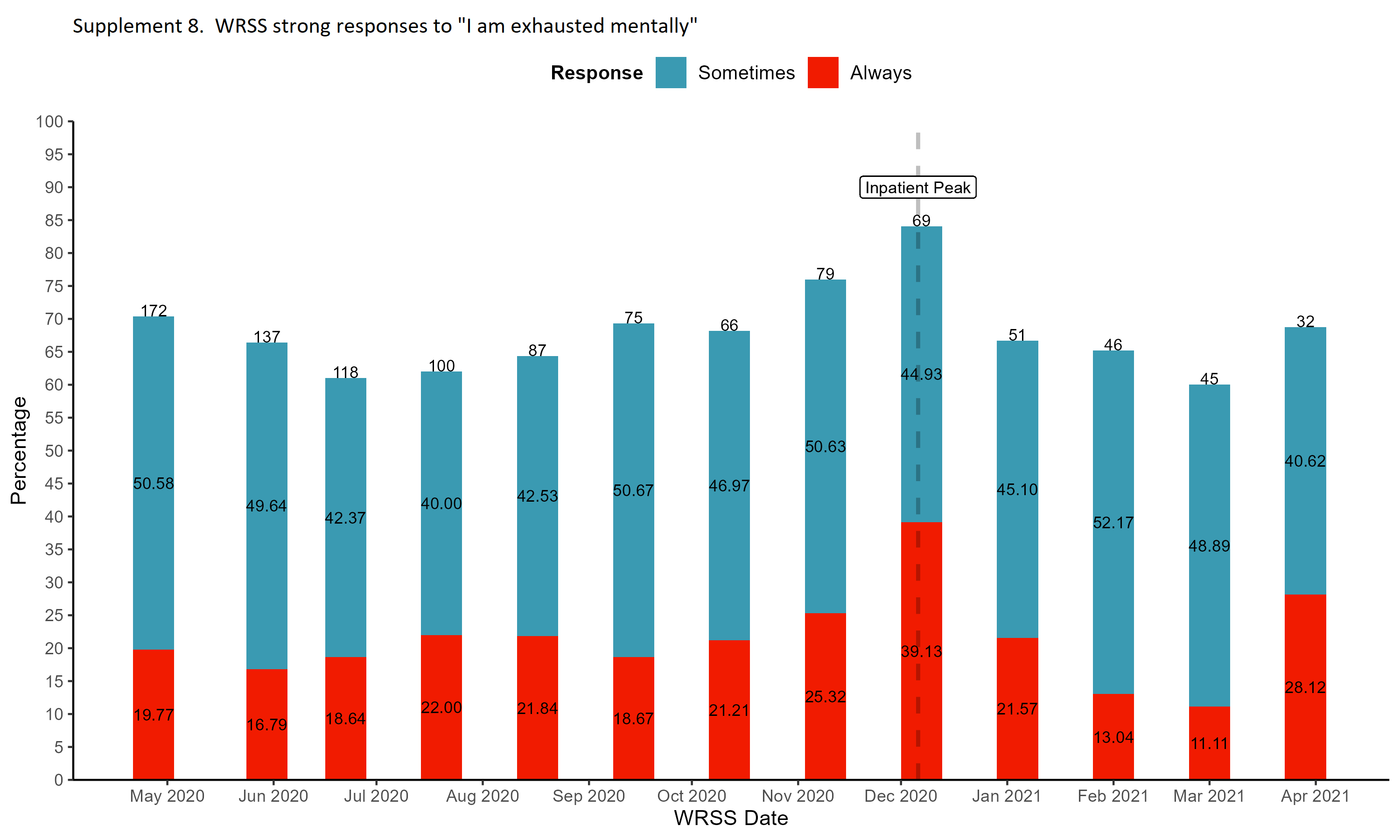

Supplement: Supplementary file 1 [file healthcare-14-01154-s001.zip › S8.tiff]

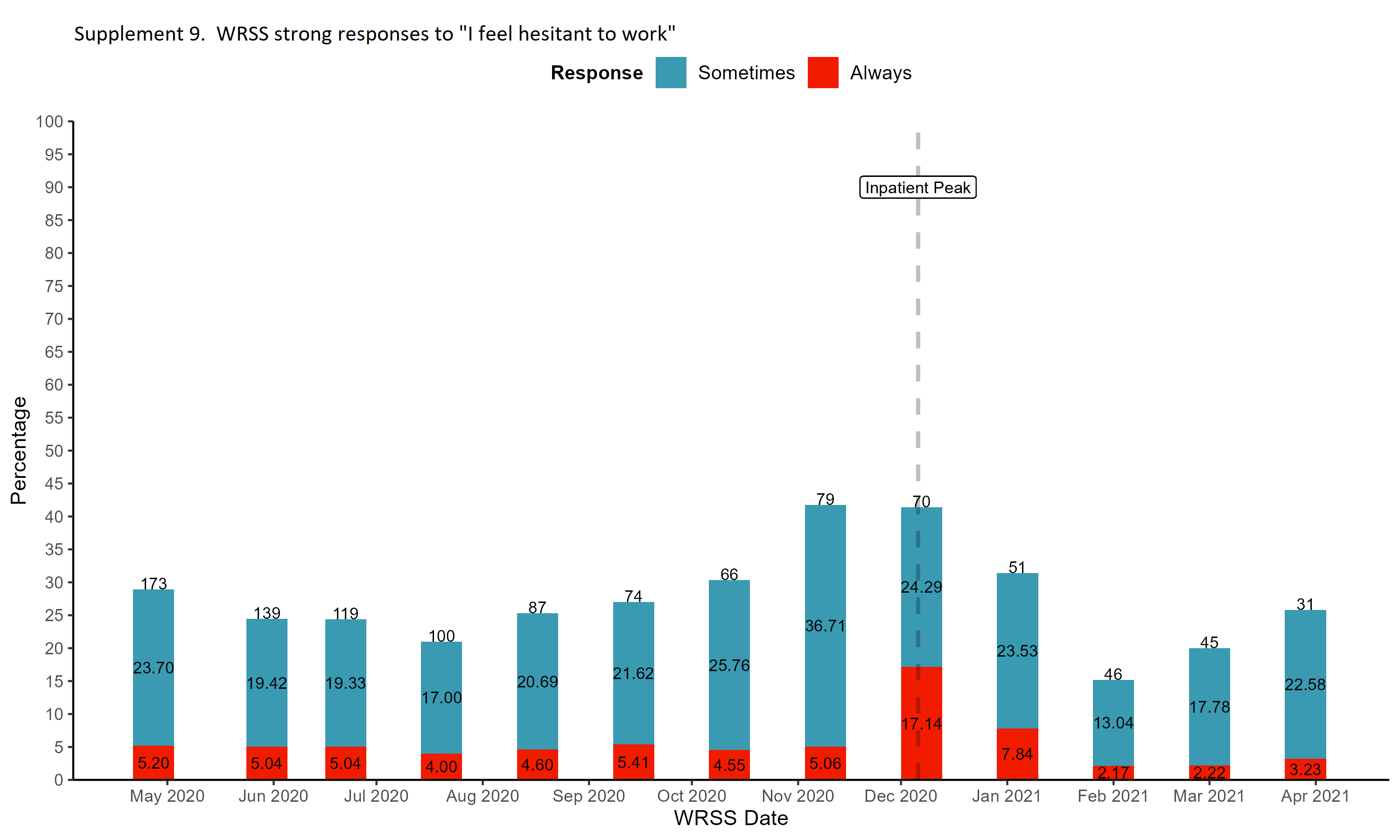

Supplement: Supplementary file 1 [file healthcare-14-01154-s001.zip › S9.tiff]
